# *Aspergillus nidulans* CkiA is an essential casein kinase I required for delivery of amino acid transporters to the plasma membrane

**DOI:** 10.1111/j.1365-2958.2012.08042.x

**Published:** 2012-04-10

**Authors:** Angeliki Apostolaki, Laura Harispe, Ana María Calcagno-Pizarelli, Ioannis Vangelatos, Vicky Sophianopoulou, Herbert N Arst, Miguel Angel Peñalva, Sotiris Amillis, Claudio Scazzocchio

**Affiliations:** 1 Institut de Génétique et Microbiologie, Université Paris-Sud (XI)UMR 8621 CNRS 91450 Orsay, France; 2 Centro de Investigaciones Biológicas, CSICRamiro de Maeztu 9, Madrid 28040, Spain; 3 Dept. of Microbiology, Imperial College London, South Kensington CampusFlowers Building, Armstrong Road, London SW7 2AZ, UK; 4 Institute of Biology, National Center for Scientific Research, Demokritos (NCSRD)Aghia Paraskevi, 15310 Athens, Greece; 5 Dept. of Genetics, University of Cambridge, Downing StreetCambridge CB2 3EH, England; 6 Faculty of Biology, University of AthensPanepistemioupolis, 15784 Athens, Greece

## Abstract

Type I casein kinases are highly conserved among Eukaryotes. Of the two *Aspergillus nidulans* casein kinases I, CkiA is related to the δ/ε mammalian kinases and to *Saccharomyces cerevisiæ* Hrr25p. CkiA is essential. Three recessive *ckiA* mutations leading to single residue substitutions, and downregulation using a repressible promoter, result in partial loss-of-function, which leads to a pleiotropic defect in amino acid utilization and resistance to toxic amino acid analogues. These phenotypes correlate with miss-routing of the YAT plasma membrane transporters AgtA (glutamate) and PrnB (proline) to the vacuole under conditions that, in the wild type, result in their delivery to the plasma membrane. Miss-routing to the vacuole and subsequent transporter degradation results in a major deficiency in the uptake of the corresponding amino acids that underlies the inability of the mutant strains to catabolize them. Our findings may have important implications for understanding how CkiA, Hrr25p and other fungal orthologues regulate the directionality of transport at the ER-Golgi interface.

## Introduction

In fungi, the uptake of most amino acids is mediated by transmembrane proteins belonging to the specific YAT (TC 2.A.3.10, yeast amino acid transporters) family. These transporters share a predicted topology comprising 12 transmembrane domains, which has been experimentally established for the *S. cerevisiæ* Gap1 (general amino acid permease, Gilstring and Ljungdahl, 2000). Members of the YAT family show, notwithstanding diverse substrate specificities, a high degree of sequence identity (André, 1995; Jack *et al*., 2000; Van Belle and André, 2001). In *S. cerevisiæ*, 18 YAT transporters have been characterized with varying degrees of detail (Jack *et al*., 2000). The genome of *Aspergillus nidulans* includes 19 predicted genes encoding transporters of this family (C. Scazzocchio, unpublished) of which two, the proline specific transporter, *prnB* (ANID_01732.3) and the dicarboxylic amino acid transporter, *agtA* (ANID_06118.3), have been functionally characterized (Sophianopoulou and Scazzocchio, 1989; Tazebay *et al*., 1995; Tavoularis *et al*., 2003; Apostolaki *et al*., 2009; Vangelatos *et al*., 2009).

The high sequence similarity extant in this family of transporters, within and across wide taxonomical boundaries, suggests that conserved structural features/mechanisms might be involved in their traffic through the endoplasmic reticulum and the Golgi, their insertion into the plasma membrane, their endocytic internalization and their delivery to the vacuole for degradation. For example, *S. cerevisiæ* Shr3p is a specific ER chaperone necessary for the efficient insertion of YAT transporters into the membrane (Ljungdahl *et al*., 1992; Kota *et al*., 2007), with orthologues characterized in *A. nidulans*, *Schizosaccharomyces pombe* and *Candida albicans* (Martínez and Ljungdahl, 2000; 2004; Erpapazoglou *et al*., 2006; Apostolaki *et al*., 2009).

The Pezizomycotina (filamentous ascomycetes) have diverged from the Saccharomycotina at least 600 million years ago (Heckman *et al*., 2001; Padovan *et al*., 2005). No systematic study of the mechanisms mediating YAT transcriptional regulation or YAT delivery to the plasma membrane has been reported for any member of this sub-phylum.

There are pointers to some differences between representatives of the Pezizomycotina and *S. cerevisiæ*. The endocytic internalization and degradation in the vacuole of many transporters, including YATs, is elicited by ammonium in both *S. cerevisiæ* (Risinger and Kaiser, 2008 and references therein) and *A. nidulans*, but the mechanism underlying this phenomenon does not seem to be identical (Apostolaki *et al*., 2009 and references therein; Gournas *et al*., 2010). The *A. nidulans* ER chaperone ShrA has a more restricted role than its yeast Shr3p counterpart (Erpapazoglou *et al*., 2006; Apostolaki *et al*., 2009). Additionally, the absence of Shr3 results in accumulation of target transporters in the ER (Ljungdahl *et al*., 1992), while the absence of ShrA results in their vacuolar localization (Apostolaki *et al*., 2009).

There are reports in early *A. nidulans* literature of several loci where mutations result specifically and pleiotropically in defective amino acid uptake (Kinghorn and Pateman, 1975; Sharma, 1984), suggesting that mechanisms of regulation and/or plasma membrane delivery common and specific to a number or all YAT proteins are operating and can be studied in this organism. In this study, we report that partial loss-of-function of an essential *A. nidulans* gene encoding a δ/ε type I casein kinase results in markedly reduced activity of most YAT proteins. To our knowledge, this is the first report implicating a casein kinase of the δ/ε isotype in the trafficking of a specific family of transporters, a finding whose significance is enhanced by recent results concerning the role of the *S. cerevisiæ* orthologue, Hrr25 in the ER-Golgi traffic (Lord *et al*., 2011).

## Results

### Three allelic mutations result in defective utilization of amino acids as nitrogen sources

This report focuses on three recessive mutations (designated, *ckiA2*, *ckiA102* and *ckiA1919*) which result specifically in impairment of growth on several amino acids as sole nitrogen sources and in resistance to toxic amino acid analogues. The three mutants were isolated by three different screening procedures, respectively the inability to utilize δ-aminovaleric acid as a nitrogen source of *puA2* strain in the presence of limiting putrescine (Herman and Clutterbuck, 1966), resistance to the toxicity of proline in a strain sensitive to semialdehyde toxicity (Arst *et al*., 1981) and resistance to toxic amino acid analogues. Details are provided in *Supplementary Experimental procedures* in the *Supporting information*. These mutations do not affect the utilization of nitrogen sources other than amino acids, such as purines (which *A. nidulans* catabolizes to ammonium), allantoin, urea, ammonium, nitrate, nitrite or acetamide. The three mutations were mapped to chromosome III, do not to complement with, and map at less than 0.2 cM, from each other (*Experimental procedures*). In conventional crosses *ckiA102* was tentatively located at 8 cM from *alX4* and 10 cM from *argB2*, the probable order being *alX–ckiA–argB*.

Interestingly, in the most recent screen (resistance to toxic amino acid analogues), only one in a total of 22 mutations defective in amino acid uptake isolated was a *ckiA* allele (*ckiA1919*). *fbaA1013*, one of the 21 mutations not allelic to *ckiA* (Apostolaki, 2003), is a down-promoter mutation in a gene encoding a fructose 1,6-biphosphate aldolase (Roumelioti *et al*., 2010). We arbitrarily selected five mutants (including *fbaA1013*), not allelic to *ckiA102*, and crossed each of them to the remaining twenty. Only two mutations were found to be possibly allelic to each other and none to *fbaA1013*. Thus, the above three *ckiA* mutations define only one of several loci where mutations leading to a pleiotropic defect in amino acid uptake map, demonstrating that this class is not saturated.

**Fig 1:**
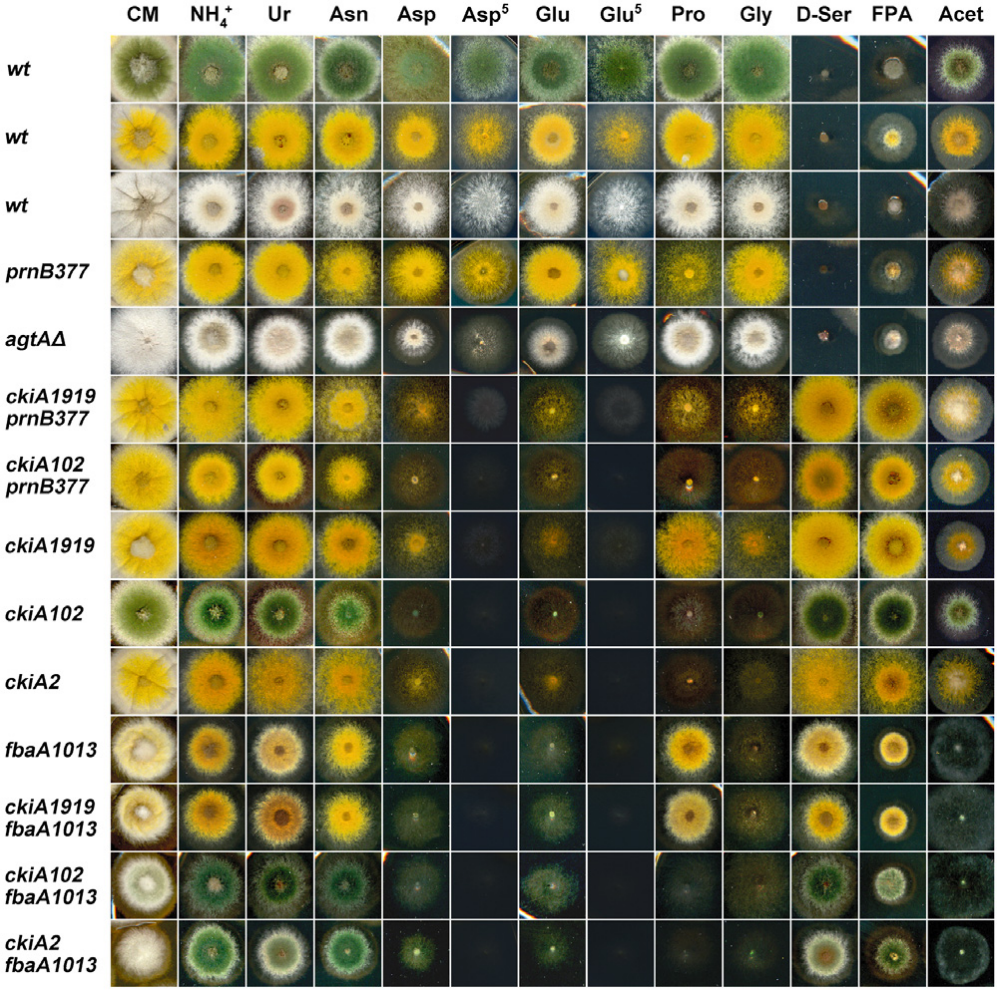
Growth phenotypes of mutant strains. The relevant genotype of each strain is shown to the left of the panel. Complete genotypes are shown in Table 1. Three wild type (*wt*) controls are included: CS2498, LH59 and CS1957. The mutant strains are from top to bottom: CAM45, VIE212, VIE181, CS1912, VIE180, CS1902, VIE174, VIE111, VIE114, VIE108, VIE113. CM: complete medium. Supplemented MM with glucose as carbon source was used with each of the following nitrogen sources: ammonium (NH_4_^+^), urea (Ur), asparagine (Asn), aspartate (Asp, Asp^5^), glutamate (Glu, Glu^5^), glycine (Gly). Asp^5^ and Glu^5^ contain the respective amino acids at 5 mM rather than at 10 mM. d-Ser and FPA indicate d-serine and dl-*p*-fluorophenylalanine on urea as nitrogen source respectively. Acet stands for acetate as a carbon source. Putative *ckiA fbaA1013* double mutants were checked by outcrossing to a wild type, with recovery of both parental classes and for the strains shown in this figure, also by confirming the presence of the relevant *ckiA* mutation after PCR amplification of the *ckiA* ORF (see text for the nature of each mutation).

While the cognate phenotypes are not identical, the three *ckiA* mutations result in strong resistance to d-serine and *p*-fluorophenyalanine, and in impaired growth on all canonical amino acids which can be utilized as nitrogen sources by *A. nidulans,* excepting glutamine and asparagine (Fig. 1), where growth is not affected. In crosses involving the *ckiA* mutations we confirmed the co-segregation of at least four phenotypes, resistance to d-serine, and to dl-*p*-fluorophenylalanine, impaired growth on glutamate and glycine, and for *ckiA102*, also impaired growth on proline, which rules out the possibility that the pleiotropic phenotypes scored result from more than one mutation. For all nitrogen sources tested a hierarchical order (of growth on amino acids as nitrogen sources) is observed in which *ckiA102* ≤ *ckiA2* < *ckiA1919* < *ckiA*^+^. The phenotypes of both *ckiA102* and *ckiA2* do not differ between 37°C and 25°C, while that of *ckiA1919* is less marked at 25°C than at 37°C (tested for utilization of glutamate and glycine and resistance to d-serine; not shown). The three alleles were also tested for their effect on the utilization of selected amino acids (glutamate, alanine, proline, phenylalanine, ornithine and threonine) as carbon sources in the presence of ammonium chloride as a nitrogen source. Ammonium chloride does not interfere with the utilization of amino acids as carbon sources. All three mutations affect amino acid utilization as carbon sources in the same hierarchical order as that described above (not shown). Finally, the mutation having the strongest phenotype, *ckiA102* also results in a somewhat restricted, less dense growth on all media tested (including complete medium and minimal medium with ammonium as nitrogen source) while *ckiA1919* has a subtle morphological phenotype most visible on complete medium, and *ckiA2* does not have an obvious morphological phenotype.

**Table 1:** *Aspergillus nidulans* strains used and constructed during this study.

Strain	Genotype	Description/references
CS2498	*pabaA1*	Wild-type reference strain
LH59	*yA2; pantoB100; riboB2*	Wild-type reference strain
CS1957	*pantoB100; wA3*	Wild-type reference strain
CS1958	*pyrG89; biA1; pyroA4; fwA1*	Apostolaki (2003)
CS2374	*pantoB100; biA1; argB2*	Apostolaki (2003)
TNO2A7	*nkuAΔ::argB; pyrG89; pyroA4; riboB2*	Nayak *et al*. (2006)
TNO2A25	*nkuAΔ::argB; pyrG89; pabaB22; riboB2; argB2*	Nayak *et al*. (2006)
CS2290	*prnB377; pabaA1; riboB2; yA2*	*prnB* deleted strain (Tazebay *et al*., 1995)
CAM45	*prnB377; pabaA1; yA2*	Tavoularis *et al*. (2001)
TpA4	*prnB::gfp; pantoB100; yA2*	Tavoularis *et al*. (2001)
CS1945	*agtAΔ::riboB; biA; pyrG89; wA3; pyroA4; riboB2*	*agtA* deleted strain (Apostolaki *et al*., 2009)
CS3095	*areA600; sb43; biA1*	*areA* null mutant (Al Taho *et al*., 1984; Kudla *et al*., 1990)
C357	*sC12; ivoA1; galA1; yA2*	Glasgow collection
C358	*sC12; alX4; palG21; dilA1; pabaA1*	Glasgow collection
C190	*cnxH5; anA1; pyrF11; luA1; yA2*	Glasgow collection
LH61	*yA; pantoB100; riboB^+^; biA1; argB2*/*yA2; pantoB100; riboB2; biA^+^; argB^+^*	Diploid, this study
CS1924	*ckiA102; prnB6; pabaA9*	*prnB6*: loss of function mutation in *prnB* (Arst and MacDonald, 1975; Arst *et al*., 1981; Sharma, 1984)
CS1904	*ckiA102; biA1*	This study
CS1947	*ckiA102; pabaA1; yA2*	This study
CS1903	*ckiA102; prnB377; pabaA1; yA2*	This study
CS1912	*ckiA102; prnB377; pabaA1; riboB2; yA2*	This study
CS1901	*ckiA102; pyrG89; pabaA1; pyroA4; yA2*	This study
CS1902	*ckiA102; riboB2; biA1*	This study
VIE178	*ckiA102; prnB::gfp; pantoB100; biA1*	This study
VIE173	*ckiA2; puA2; biA1; fwA1*	This study
CS1959	*ckiA2; yA2; puA2*	This study
VIE174	*ckiA2; pyroA4; yA2*	This study
VIE175	*ckiA2; pabaA1*	This study
VIE181	*ckiA1919; prnB377; pabaA1; riboB2; yA2*	This study
VIE179	*ckiA1919; pyrG89; pabaA1*	This study
VIE180	*ckiA1919; pabaA1; pantoB100; yA2*	This study
VIE112	*fbaA1013; pabaA1; prnB377; riboB2; yA2*	Roumelioti *et al*. (2010)
KR1	*fbaA1013; pyrG89; pabaA1; pantoB100; yA2*	Roumelioti *et al*. (2010)
VIE116	*fbaA1013; pabaA1; pyrG89; pantoB100; yA2*	Roumelioti *et al*. (2010)
VIE111	*fbaA1013; pabaA1; riboB2; yA2*	This study
VIE107	*ckiA102; fbaA1013; pabaA1; yA2*	This study
VIE108	*ckiA102; fbaA1013; riboB2*	This study
VIE113	*ckiA2; fbaA1013; pabaA1; pantoB100*	This study
VIE114	*ckiA1919; fbaA1013; pantoB100; yA2*	This study
VIE115	*ckiA1919; fbaA1013; riboB2; yA2*	This study
LH121	*agtA::HA::AFpyrG; pyroA4*	Apostolaki *et al*. (2009)
CAM13	*ckiA102; agtA::HA::AFpyrG; riboB2; nkuAΔ::argB; pyrG89*	This study
AMC264	*ckiA2; agtA::HA::AFpyrG; pyroA4*	This study
LH127	*ckiA1919; agtA::HA::AFpyrG; pabaA1*	This study
LH115	*agtA::gfp::AFpyrG; pabaA1; pyroA4; pyrG89; yA2*	Apostolaki *et al*. (2009)
AMC129	*cki102; agtA::gfp::AFpyrG; yA2; pabaA1; pyrG89*	This study
AMC314	*ckiA2;agtA::GFP::AFpyrG; pabaA1; yA2*	This study
CAM51	*cki1919; agtA::GFP::AFpyrG; pyroA4*	This study
VIE050	*thiAp:ckiA; agtA::gfp::AFpyrG; pabaA1; yA2*	This study
VIE051	*thiAp::FLAG::ckiA; agtA::gfp::AFpyrG;pabaA1; yA2*	This study
VIE047	*thiAp::gfp::ckiA::AFriboB; pyrG89 nkuAΔ::argB; pyroA4; riboB2*	This study
VIE172	*ckiA::gfp::AFpyrG; pabaB22; riboB2*	This study

All three *ckiA* mutations impair the utilization of β-alanine and δ-aminovaleric acid, while not affecting the utilization of γ-aminobutyric acid (GABA). Most strongly affected is the utilization of aromatic and dicarboxylic amino acids and glycine. Indeed, the two more extreme *ckiA* mutations affect growth on 10 mM aspartate and glutamate more strongly than a deletion of the specific *agtA* transporter gene (Apostolaki *et al*., 2009), which implies that both dicarboxylic amino acids can be taken up by at least a second, lower-affinity transporter.

**Fig 2:**
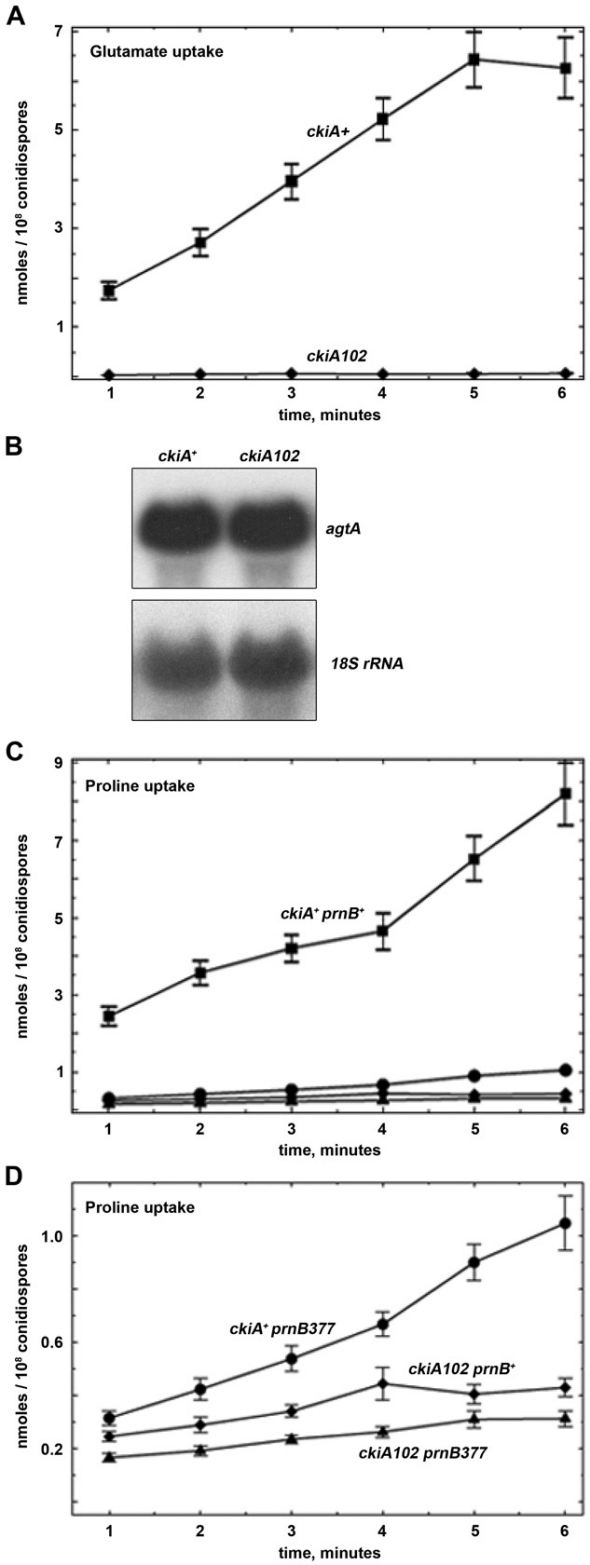
*ckiA102* strains are defective in amino acid uptake. A. Glutamate uptake by *ckiA*^+^ (CS2498, squares, bars standard errors) and *ckiA102* (CS1947, diamonds, error bars not shown as the uptake is almost nil) strains. B. Northern blot showing the expression of *agtA* in germlings pre-grown in the same conditions as for the uptake measurements (see *Experimental procedures*). C and D. Proline uptake by *prnB^+^ckiA^+^* (CS2498, squares), *prnB377* (CAM45, circles)*, ckiA102* (CS1947, diamonds) and *ckiA102 prnB377* (CS1903, triangles, the latter two not clearly separated in C) strains. (D) Graph shows the same data as (C) for the three mutant strains, with an expanded scale in order to magnify the differences between them. Bars represent standard errors.

Null mutations in *prnB*, encoding the major proline permease, allow residual growth on proline as sole nitrogen source and strongly reduce proline uptake (Tazebay *et al*., 1995; Tavoularis *et al*., 2003; see Figs 1 and 2 and below), which has been interpreted as proline being taken up also by at least one other transporter. The utilization of proline is strongly affected by *ckiA102* and *ckiA2* and very mildly impaired by *ckiA1919*, the *ckiA^-^* phenotype being partially additive with that of the null *prnB* allele (Fig. 1; additivity not tested for *ckiA2*). This implies that *ckiA102* and *ckiA2* mutants showing markedly impaired utilization of proline are defective in the activity of both PrnB and the alternative proline uptake system.

Double mutants carrying each of the three *ckiA* alleles and *fbaA1013* were constructed (Fig. 1). The results of these tests are consistent with *ckiA* and *fbaA* not acting sequentially in the same pathway. *ckiA* alleles do not affect growth on acetate, a characteristic of the *fbaA1013* mutation (Roumelioti *et al*., 2010). Double mutants show restricted growth on complete medium, a phenotype somewhat more restricted than that of *fbaA1013*, more clearly shown for the *ckiA2 fbaA1013* double mutants. On all nitrogen sources tested, the double mutant shows the phenotype of the more extreme parent. This is most noticeable on proline, where *fbaA1013* strains are hardly affected but *ckiA2 fbaA1013* and *ckiA102 fbaA1013* are as affected as the *ckiA* parent. Weak additivity can be seen for growth on glutamate and aspartate at 10 mM for the *ckiA2* and *ckiA102 fbaA1013* double mutants. The *ckiA fbaA1013* double mutants show more restricted growth on d-serine and *p*-fluorophenylalanine than either parent strain, this possibly due to the restricted colony morphology seen on all media for strains carrying *fbaA1013*.

### *ckiA102* results in defective glutamate and proline uptake

The pleiotropic phenotypes of *ckiA* mutants are most readily explained by impaired amino acid uptake. Consistent with this interpretation is the fact that we have been unable to isolate *ckiA102 argB2* and *ckiA102 leuA2* recombinants (*argB2* and *leuA2* result in arginine and leucine auxotrophies respectively) in crosses where these markers were segregating. The three *ckiA* mutations result in a drastic impairment of dicarboxylic amino acid utilization and *ckiA2* and *ckiA102* also in significant impairment of proline utilization. Thus, we tested the activities of the well characterized AgtA (Apostolaki *et al*., 2009) and PrnB transporters (Tazebay *et al*., 1995; Tavoularis *et al*., 2003 and references therein) to investigate the basis of these defects. Figure 2A shows that a strain carrying *ckiA102* is unable to take up glutamate at the concentration tested. Figure 2C and D shows the same strain being more drastically affected for the uptake of proline than a *prnB* deletion mutant, which implies that *ckiA102* affects both the specific proline transporter, PrnB, and alternative proline transporter(s). Partial additivity is seen for the *ckiA102 prnB377* double mutant (Fig. 2D).

### Identification of the *ckiA* gene

The *ckiA* gene was identified by complementation of the *ckiA102* mutation with a genomic library constructed in a self-replicating vector (Osherov and May, 2000) and with pools from the minimal ordered compressed cosmid library of the third chromosome of *A. nidulans* (see *Experimental procedures*). From the first library, the smallest plasmid insert that complemented the *ckiA102* mutation contained the whole open reading frame of ANID_04563.1 (http://www.broadinstitute.org/annotation/genome/aspergillus_group/MultiHome.html) encoding a casein kinase I, together with 322 bp of its upstream region and a partial sequence of the adjacent ANID_04562.1 gene. *ckiA102*-complementing cosmid L12G07 cross-hybridized with the above insert and one subclone containing ANID_04563.1 plus 1053 bp upstream and 595 bp downstream of the ORF also complemented *ckiA102* (see *Experimental procedures*). Moreover, ANID_04563.1 is located in contig 78, chromosome III, between ANID_04409.1 (*argB* in contig 76) and ANID_04603.1 (*alX*, in contig 79; Hamari *et al*., 2009) in agreement with the position determined for *ckiA* in the genetic map. These findings and the identification within ANID_04563.1 of the three *ckiA* mutations (see below) established unambiguously that *ckiA* is ANID_04563.1.

The predicted ANID_04563.1 ORF encodes 367-amino-acid residues and is interrupted by two introns near the 5′ end of the coding region. An almost complete cDNA sequence was obtained (M. Billini and V. Sophianopoulou, unpublished; accession number HQ 661153) that experimentally confirmed the presence and position of these introns and established the position of the initiation codon. Northern blots showed an mRNA of about 2.5 kb in both *ckiA^+^* and *ckiA102* strains (not shown). The mRNA steady state levels are nearly identical in mycelia grown on either ammonium or glutamate as nitrogen sources, and this throughout the morphological transition from isotropic growth to young hyphae. The *ckiA* mRNA is also present in ungerminated conidiospores (Fig. S1).

### *ckiA* encodes a δ/ε casein kinase: phylogeny of fungal casein kinases I

**Fig 3:**
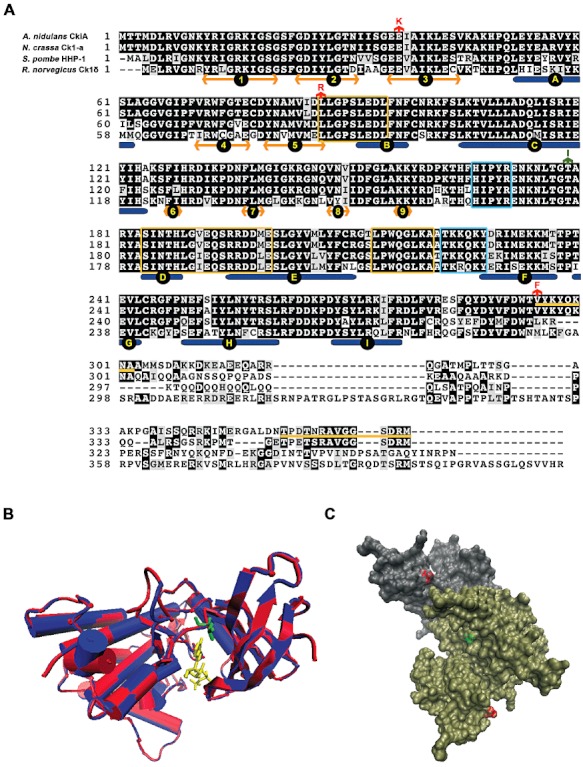
A. Alignment of *A. nidulans* CkiA with selected orthologues (see text). Alignment carried out with MUSCLE and visualized with box-shade. Under the alignment the succession of β-sheets and α-helices as deduced from known structures is shown (Longenecker *et al*., 1996). Double headed golden arrows, β-sheets (numbered from 1–9), rounded-ended blue rectangles, α-helixes (A to I). The sequences boxed in yellow represent the signature motifs of casein kinase I proteins (see text), the sequences boxed in cyan, the kinesin homology domain and downstream from it the SV40 T-antigen nuclear localization sequence (Gross and Anderson, 1998). The yellow lines between the sequences of the *A. nidulans* and *N. crassa* orthologues show sequences that are conserved in the Pezizomycotina but not among other ascomycetes (see text). The three amino acid substitutions described in this article are indicated by red arrows above the *A. nidulans* CkiA sequence. In green we indicate the position and mutant change of the *hrr25-2* (*rst2-1*) suppressor of *S. cerevisiæ* (Murakami *et al*., 1999; see *Discussion*). B. Model of the CkiA protein. In red, CkiA, Val8-Thr294, in blue *S. pombe* Cki1 Asn6-Leu298 (PDB 1cnsA). In yellow ATP molecule. The substrate lies in a groove between the β-sheet lobe and α-helix lobe of the molecule. Leucine 87 is shown in green. We show the comparison with the *S. pombe*γ-like homologue, rather than the closer rat δ-homologue (Longenecker *et al*., 1996; see Fig. 4) as only the former has been crystallized with the bound ATP (Xu *et al*., 1995). C. Surface representation of the rat casein kinase I δ dimer (Longenecker *et al*., 1996) showing the surface residues discussed in this article. Subunit A in grey, subunit B in tan. In red Glu 34, corresponding to Glu 37 of CkiA, in green Thr 176, corresponding to Thr 176 of Hrr25. Modelling with I-Tasser, images made with VMD/NAMD/BioCoRE/JMV/other software support. VMD/NAMD/BioCoRE/JMV/ is developed with NIH support by the Theoretical and Computational Biophysics group at the Beckman Institute, University of Illinois at Urbana-Champaign.

CkiA shows a striking amino acid sequence identity with mammalian δ/ε and related fungal casein kinases I as illustrated in Fig. 3. Two structures of close CkiA relatives have been solved, those of *S. pombe* Cki1 (Xu *et al*., 1995) and of *Rattus norvegicus* Casein kinase 1-δ (Longenecker *et al*., 1996). The structures comprise two lobes, which together form the catalytic domain. The amino-terminal lobe contains five antiparallel β-sheets interrupted by an α-helix (Fig. 3A), while the carboxy-terminal lobe consists in a succession of α-helixes. CkiA can be readily modelled on these structures (Fig. 3B), the predicted structure showing an identical succession of β-sheets and α-helices to that seen in the solved structures (Fig. 3A; see below and *Discussion*). The three signature motifs of casein kinases I (Longenecker *et al*., 1996) are conserved in CkiA. The first, LLGPSLEDL, is the linker peptide between the β and α lobes of the protein. The second, SINTHLGIEQSRRDDLE, spans the α−D and α−E helixes. It contains in CkiA two conservative substitutions (in the residues underlined above) shared with other fungal orthologues (Fig. 3). This motif comprises the SIN (Ser-Ile-Asn) sequence that in all type I casein kinases replaces the Ala-Pro-Glu sequence present in protein kinases of other families. The third motif LPWQGLKA, fully conserved in CkiA, is comprised in the loop between helices α−E and α−F. A putative nuclear entry signal, typical of casein kinases I, and a kinesin–tubulin interaction domain complete the inventory of conserved signatures (Fig. 3A).

**Fig 4:**
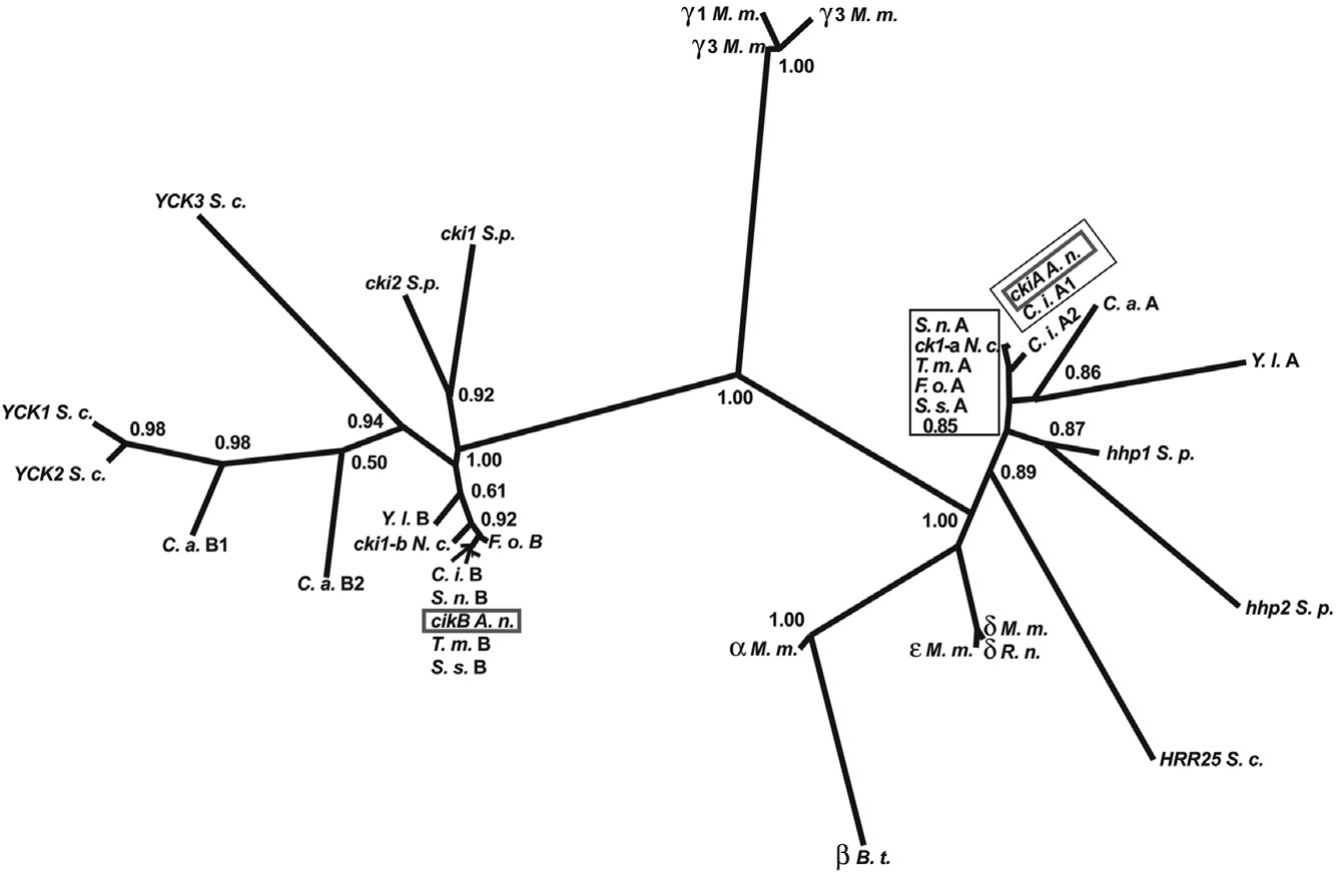
Phylogeny of the ascomycete casein kinase I proteins. This tree includes casein kinases I from representative species of the ascomycetes. All the isoforms of *Mus musculus* (*M. m.*) are also included, together with the unique isoform β of *Bos taurus* (*B. t.*) and the isoform δ of *Rattus norvegicus* (*R. n.*) mentioned in various contexts in the text. Characterized genes of *A. nidulans*, *N. crassa, S. cerevisiæ* and *S. pombe* are indicated by their standard genetic symbols followed by the abbreviation of the species. Other ORFs are indicated by the following convention: A two letter abbreviation of the species followed by ‘A’ for the homologues of *ckiA*, *HRR25*, *hhp1* and *hhp2*, which never have a palmitoylation signal in the their carboxy-terminus, and ‘B’ for the homologues of *YCK* which always carry such a signal. When more than one ‘A’ or ‘B’ homologue is present for a given species, this indicated by a number following the letters A or B. The ascomycete species included are: Taphrinomycotina: *Schizosaccharomyces pombe* (*S. p.*); Saccharomycotina, Saccharomycetales: *Saccharomyces cerevisiæ* (*S. c.*), *Candida albicans* (*C. a.*), *Yarrowia lypolitica* (*Y. l.*). Pezizomycotina: Eurotiales, *Aspergillus nidulans* (*A. n.*), Onygenales, *Coccidioides immitis* (*C. i.*), Sordariales, *Neurospora crassa* (*N. c*.), Hypocreales, *Fusarium oxysporum* (*F. o.*), Helotiales, *Sclerotina sclerotium* (*S. s.*), Pleosporales, *Stagonospora nodorum* (*S. n.*), Pezizales, *Tuber melansporum* (*T. m.*). ORF sequences were obtained from the appropriate databases (see *Supplementary Experimental procedures* for accession numbers) and aligned with T-Coffee. The phylogenetic tree was redrawn from an original PhyMyl (Guindon *et al*., 2010) tree obtained after curation of the alignment with G-blocks and visualized with Drawtree (see *Experimental procedures*). The decimals in the nodes indicate the values of the Approximate Probability Ratio Test (aLTR; Anisimova and Gascuel, 2006). For clarity's sake some of the values for the minor nodes are not included. The boxed homologues are not separated in this tree, the 0.85 value refers to the aLTR separating the two boxed branches containing the Pezizomycotina homologues in clade ‘A’. The non-boxed homologues in a column in the ‘B’ clade are separated by extremely short branches, they correspond to up to bottom to the five short branches, left to right. Note the very high branch support for all the major nodes. The two *A. nidulans* genes are highlighted by a grey box. Note the striking conservation of sequences within the Pezizomycotina, including *Tuber melanosporum*, a basal species (Spatafora *et al*., 2006; see Fig. 4), which has diverged from the crown orders of the Pezizomycotina (including all the other Pezizomycotina represented in the figure) about 300 million years ago (Berbee and Taylor, 2007; Lucking *et al*., 2009).

A phylogeny of the casein kinases I proteins is shown in Fig. 4. The class I casein kinases of the ascomycetes fall into two clades. In all available Pezizomycotina proteomes one homologue (exceptionally two; see Fig. 4) of each clade is present. One clade includes the Yck proteins of *S. cerevisiæ* and comprises proteins that all have a palmitoylation motif in their carboxy-terminus. This motif is also present in membrane-anchored γ-mammalian Casein kinase I isoform (Davidson *et al*., 2005). The *S. cerevisiæ* enzymes are anchored to the plasma (Yck1 and Yck2) or vacuolar (Yck3) membranes via a covalently bound fatty acid (Vancura *et al*., 1994; Roth *et al*., 2002; Sun *et al*., 2004). The homologous *A. nidulans* ANID_05757.1, designated *ckiB* (see Fig. 4) is also membrane-bound (S. Amillis, T. Schinko, J. Strauss and C. Scazzocchio, unpublished).

Members of the second clade are proteins related to the metazoan δ and ε casein kinases, which do not carry a palmitoylation signal. This group includes the Hhp1 and Hhp2 paralogues of *S*. *pombe*, Hrr25p of *S. cerevisiæ*, CK1a of *N. crassa* and CkiA of *A. nidulans.* Two conserved carboxy-terminal amino acid sequence motifs are characteristic of proteins of the Pezizomycotina, being absent in the homologues of other fungi (see Fig. 3 for a comparison with the *S. pombe* orthologue). The carboxy-terminal motif VYKYQKNA is completely conserved in all available protein sequences of the Pezizomycotina (the Thr preceding this sequence is conserved also in other ascomycetes, but not in the mammalian isotype δ sequences). The VYKQKNA motif follows a universally conserved sequence, which in the crystal structure of the rat enzyme is located downstream of helix α−I and is structurally disordered (see Fig. 3A). This motif is linked by a less conserved K/N rich sequence to the most carboxy-terminal conserved motif, TP(D/E)T(N/S)RAVGGSDRM specific of δ/ε-like casein kinases of the Pezizomycotina (see the comparison of CkiA with the *N. crassa* orthologue in Fig. 3A). In addition to these conserved motifs, the *S. cerevisiæ* CkiA orthologue Hrr25p carries a long sequence in its carboxy-terminus which is absent from the *S. pombe* homologues and from all the Pezizomycotina proteins (not shown). Breitkreutz *et al*., (2010, *Supporting information*) identified within this non-conserved Hrr25 sequence two phosphorylated peptides, located between residues 310 and 388. Finally, it is worth noting that there is a striking amino acid sequence conservation both within the *ckiA*-like (δ/ε-related) and *ckiB*-like (possibly γ-related) clades of all the Pezizomycotina which contrasts with the sequence divergence seen in other ascomycetes (Saccharomycotina, Taphrynomycotina), even between paralogues of the same species (see Fig. 4).

### Identification of the *ckiA* mutations

The *ckiA* gene of strains carrying each of the three mutant alleles was sequenced. *ckiA2* is a G to A transition in nucleotide 109 (numbering from the A of the ATG initiation codon), resulting in a Glu37 to Lys substitution; *ckiA1919* is a T to G transversion in nucleotide 260 resulting in a Leu87 to Arg substitution; *ckiA102* is a G to T transversion in nucleotide 838 resulting in a Val295 to Phe substitution. The mutated residues are indicated in Fig. 3. Glu37 is conserved across all species in the δ/ε kinases (but not in the Yck/ANID_05757.3 fungal clade, nor in mammalian class I casein kinases outside the δ/ε clade). Leu87 is universally conserved. In the *S. pombe* CkI, it is one of the residues that form the hydrophobic pocket where the adenine ring of the ATP is lodged into the solved structure (Xu *et al*., 1995; see *Discussion*; Fig. 3B). Val295 is the N-terminal residue of the most carboxy-terminal conserved motif specific for the δ-like casein kinases of the Pezizomycotina.

### *ckiA* is an essential gene

We attempted to delete *ckiA* by replacement of its coding region by a ‘split’*pantoB* marker in a *pantoB100* strain and, when *nkuΔ* strains became available (Nayak *et al*., 2006), by the *A. fumigatus riboB* marker to complement *riboB2*. Only heterokaryotic transformants (and in the *nkuA^+^* strain also heterologous insertions) were obtained, indicating that *ckiA* is essential (O'Donnell *et al*., 1991; Osmani *et al*., 2006). A recent systematic initiative to delete all *A. nidulans* kinases by insertion of the *A. fumigatus pyrG* marker confirmed that *ckiAΔ* can be rescued in heterokaryons but not in homokaryons (Osmani and de Souza, http://www.fgsc.net/Aspergillus/KO_Cassettes.htm).

We also demonstrated that the lethal *ckiAΔ* phenotype is recessive in a heterozygous diploid and that *ckiAΔ* strains cannot be recovered by haploidization: diploid strain LH61 (relevant markers, *yA^+^ pantoB100 argB^+^*/*yA2 pantoB100 argB2*), homozygous for *pantoB100* and heterozygous for makers on chromosomes I (*yA*) and III (*argB*) (*ckiA* is located on chromosome III) was transformed with the *ckiAΔ::pantoB^+^* deletion cassette. Five pantothenic acid prototrophs analysed by Southern blots were all shown to be heterozygous for the *ckiA^+^* and the *ckiAΔ* alleles (Fig. S2). Two of these, T12 and T15, were haploidized. No *pantoB^+^* (i.e. *ckiAΔ::pantoB^+^*) strain was obtained among a total of 150 haploid sectors recovered. The 100 haploid sectors arising from T12 were all *pantoB100* and *argB^+^* whereas the 50 haploid strains isolated from T15 were all *pantoB100* and *argB2*, indicating that the *ckiAΔ* deletion event in T12 had occurred in coupling with *argB2* while the one in T15 had occurred in coupling with *argB^+^.* Thus these results also establish that the failure to recover *ckiAΔ* strains is due to its lethality rather than to a possible co-lethality of *ckiAΔ* and *argB2.* The chromosome I *yA* marker segregated randomly in the haploid sectors. In summary, several independent lines of evidence establish beyond question that *ckiA* is an essential gene. This is of some importance as earlier reports claimed that *S. cerevisiæ HRR25* is not essential (Hoekstra *et al*., 1991; Wang *et al*., 1996; Murakami *et al*., 1999), in stark contradiction with a recent article (Lord *et al*., 2011). The strain deleted for both the two *S. pombe* paralogues is viable (Dhillon and Hoekstra, 1994; Bimbó*et al*., 2005) but the *N. crassa* orthologue seems to be essential (Görl *et al*., 2001, systematic deletion data in http://www.dartmouth.edu/~neurosporagenome/knockouts_completed.html).

### CkiA localizes both to the cytoplasm and nucleus

**Fig 5:**
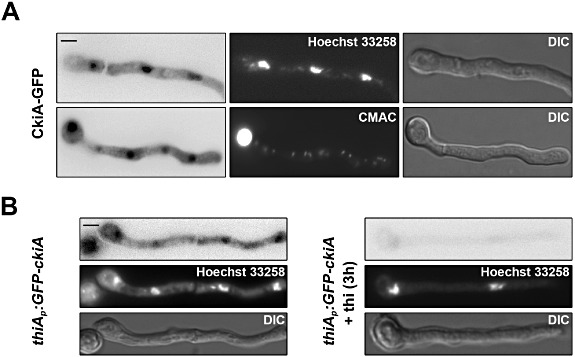
Intracellular localization of CkiA–GFP. A. Localization of CkiA–GFP in germlings. Two germlings are shown, the top one is counterstained with Hoechst 33258, the bottom one with CMAC. Germlings of strain VIE172 grown in MM for 16 h on urea as sole nitrogen source. B. Epifluorescence detection of GFP-CkiA driven by the *thiA* promoter in germlings (strain VIE047) grown in MM for 14 h on proline as sole nitrogen source and then shifted to proline in the absence and presence of thiamine (thi) for the additional time indicated. Counterstaining with Hoechst 33258 is also shown. Scale Bars: 5 µm.

A *ckiA–gfp* fusion was constructed by gene replacement, resulting in a strain where the fusion protein, expressed at physiological levels, is the only source of *ckiA*. This strain is wild type for growth on all amino acids tested, is as sensitive as the wild type to d-serine and *p*-fluorophenylalanine and does not show the somewhat restricted growth shown by the *ckiA102* strains on utilizable nitrogen sources, leading us to conclude that the fusion protein is fully functional. Both epifluorescence (Fig. 5A) and confocal microscopy (not shown) showed that CkiA–GFP is present throughout the cytoplasm and in the nuclei. We never observed any association of CkiA–GFP to the membrane, the septum or to the tip of the growing hyphæ. This intracellular distribution was identical on all nitrogen sources tested (ammonium, urea, glutamate, aspartate, proline, glycine, not shown) and did not change either with the morphological switch from conidia to hyphae (results not shown). However, it is worth noting that the protein was detectable in quiescent conidia, where we had also detected *ckiA* mRNA (see above; Fig. S1). The localization of CkiA–GFP in conidia and conidiophores is shown in Fig. S3.

### Phenotype of a *ckiA* conditional mutation

**Fig 6:**
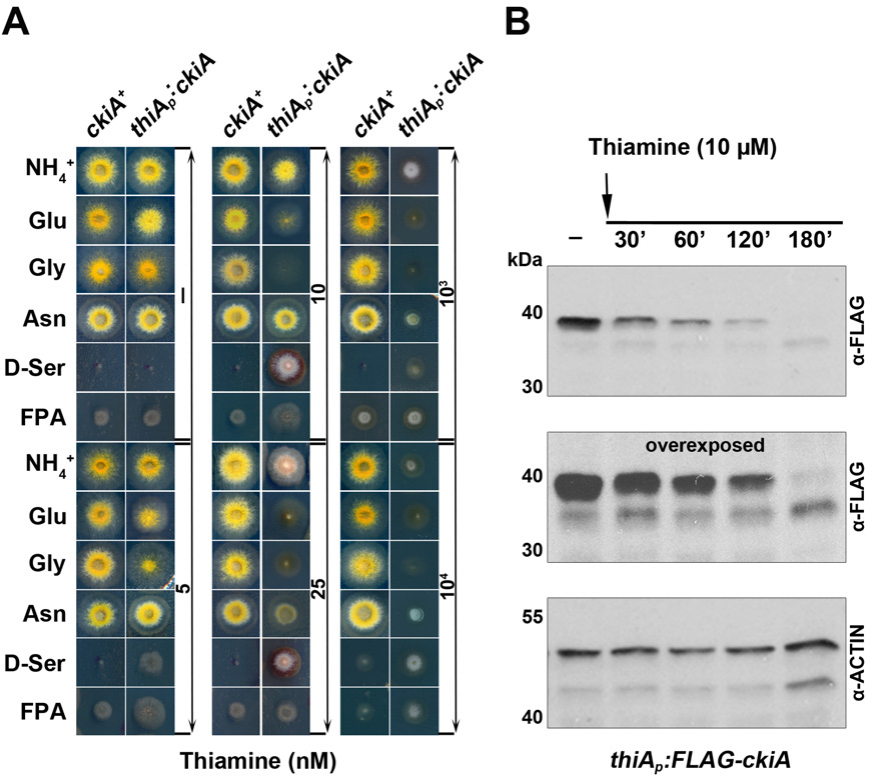
Phenotype of a *ckiA* conditional mutation. A. Growth tests of strains LH59 (wt) and VIE050 (*thiAp–ckiA*) carried out on supplemented MM with nitrogen sources or inhibitors indicated as in Fig. 1. Only relevant genotypes are shown. For complete genotypes see Table 1. Adjacent to each panel, thiamine concentrations in nM. Growth tests were carried out at 37°C and pH 6.8 for 48 h. B. Depletion of the FLAG-CkiA protein after the addition of 10 µM thiamine. Mycelia were grown for 14 h on ammonium as sole nitrogen source before addition of thiamine.

The recessivity of the classical *ckiA* mutations strongly indicates that they result from a partial loss-of-function. To buttress this proposal and to investigate the effects of CkiA depletion we constructed a conditional *ckiA* allele by placing the *ckiA* gene under the control of the *thiA* promoter, which is repressible by thiamine (M. Mathieu, A. Rincón, and C. Scazzocchio, unpublished; Calcagno-Pizarelli *et al*., 2011). We constructed strains carrying the *gfp*-tagged *agtA* gene (Apostolaki *et al*., 2009), together with the *thiA^p^–ckiA* transgene, with or without a FLAG epitope tag in the amino-terminus of CkiA. Additionally, we constructed a *thiA^p^*–*gfp*–*ckiA* strain carrying the *gfp* sequence in the amino-terminus of *ckiA*, which shows a repression-sensitive localization of the protein to cytoplasm and nucleus (Fig. 5B). Figure 6A shows the phenotype of the *thiA^p^::ckiA agtA–gfp* strain (identical phenotypic responses were seen for the other strains, not shown). In the presence of as little of 5 nM thiamine, a diminution of growth on glutamate, aspartate and glycine as nitrogen sources is noticeable together with a moderate resistance to d-serine and *p*-fluorophenylalanine (Fig. 6A; not shown for aspartate). At 25 nM thiamine, the phenotype of this strain is very similar to that of *ckiA2* and *ckiA102*, but in addition, a restricted growth on other nitrogen sources (such ammonium and nitrate, only shown for ammonium) is seen, as expected from the lethal phenotype of the deletion. The effect of thiamine on ammonium (another utilizable nitrogen source) is steadily more pronounced with increasing thiamine concentrations, but no complete inhibition of growth is seen even at 100 µM thiamine (shown for 10 µM in Fig. 6A), indicating that the repression of the *thiA* promoter is not complete. We used the *thiA^p^–flag–ckiA* strain to determine experimentally the degree of CkiA downregulation by Western blots. These experiments showed that while thiamine repression was efficient and CkiA levels rapidly (within 1–2 h) decreased after shifting cells to thiamine, even after 3 h of repression by 10 µM thiamine, a faint band CkiA::FLAG could be seen in Western blots (Fig. 6B), in agreement with growth tests indicating that repression is incomplete.

### *ckiA* mutant alleles and CkiA depletion result in miss-routing of amino acid transporters into the vacuole

**Fig 7:**
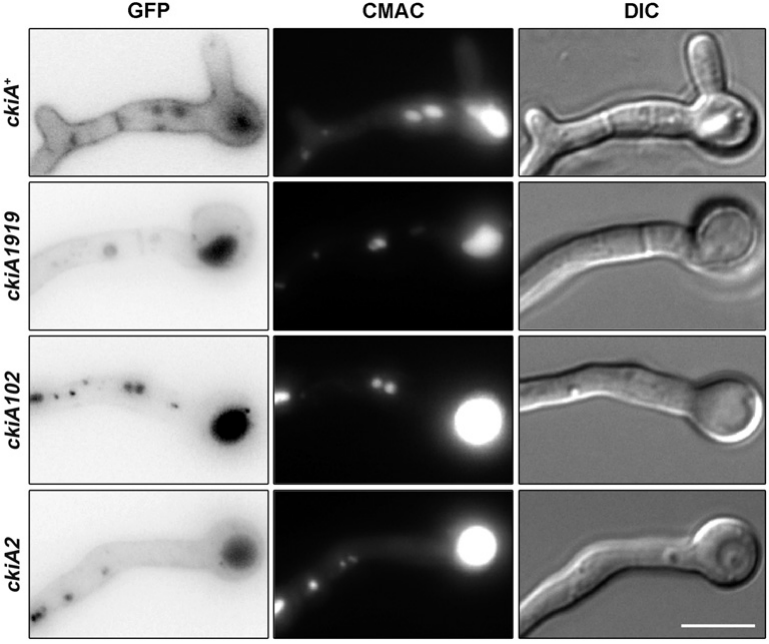
Localization of AgtA–GFP in *ckiA* mutants. Epifluorescence images of AgtA–GFP in strains carrying each of the three *ckiA* alleles (left panel). CMAC and differential interference contrast microscopy (DIC) images are shown on the middle and right panels respectively. Strains AMC132, CAM51, AMC314 and AMC129 were grown in MM containing GABA as nitrogen source for 16 h at 25°C. Scale Bar (5 µm) shown to the right of the DIC panel.

Northern blots demonstrated that steady state mRNA of *agtA* (encoding the specific dicarboxylic amino acid transporter of *A. nidulans*; Apostolaki *et al*., 2009) were not affected by the phenotypically strongest mutation, *ckiA102* (Fig. 2), despite the fact that the mutation markedly impairs growth on dicarboxylic amino acids (Fig. 1). Thus, we further investigated the effect of the *ckiA* mutations on levels and subcellular localization of GFP-tagged AgtA. Plasma membrane levels of AgtA are exquisitely regulated through transcriptional and post-transcriptional mechanisms (Apostolaki *et al*., 2009). In cells cultured on GABA, AgtA is efficiently synthesized and delivered to the plasma membrane. However, if these cells are shifted to ammonium (the preferred nitrogen source for *A. nidulans*), *agtA* transcription is efficiently shut-off and the pool of plasma membrane-resident permease is internalized by endocytosis, sorted into the multivesicular body (MVB) pathway and delivered to the vacuole for degradation (Apostolaki *et al*., 2009; Abenza *et al*., 2010; Calcagno-Pizarelli *et al*., 2011). As a result of the balance between these two mechanisms, in *ckiA^+^* strains cultured on GABA, AgtA–GFP localizes mainly to the cell membrane and secondarily to the vacuole (Apostolaki *et al*., 2009; Fig. 7, top panels). In contrast, in strains carrying *ckiA102*, AgtA–GFP is present exclusively in the vacuole, resembling the wild-type situation after shifting cells to ammonium. *ckiA2* and *ckiA1919* had a milder effect: some residual fluorescence of AgtA–GFP can be seen in the membrane of *ckiA2* and even more clearly of *ckiA1919* strains (Fig. 7). Thus, the severity of the phenotypes seen in growth tests inversely correlates with the relative AgtA levels at the plasma membrane (Figs 1 and 7).

**Fig 8:**
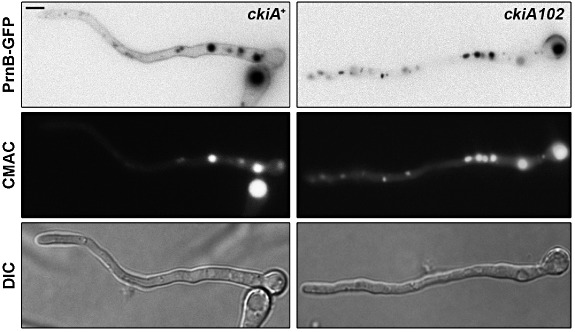
Localization of PrnB–GFP in a *ckiA102* mutant. Germlings of strain TpA4 and VIE178 were grown on ammonium as nitrogen source for 12 h and then shifted to MM containing 10 mM proline for additional 4 h. Both counterstaining with CMAC and DIC images are shown. Scale Bars: 5 µm.

We also checked the localization of the proline transporter PrnB (see Figs 1 and 2), which is also delivered from the plasma membrane to the vacuole when cells cultured on proline are shifted to ammonium (Apostolaki *et al*., 2009). In strains carrying *ckiA102* cultured on proline PrnB–GFP is localized exclusively to the vacuole (Fig. 8). All the above data strongly indicate that miss-routing of plasma membrane transporters to the vacuole is the common defect underlying the pleiotropic effects of *ckiA^-^* mutations on the utilization of amino acids.

**Fig 9:**
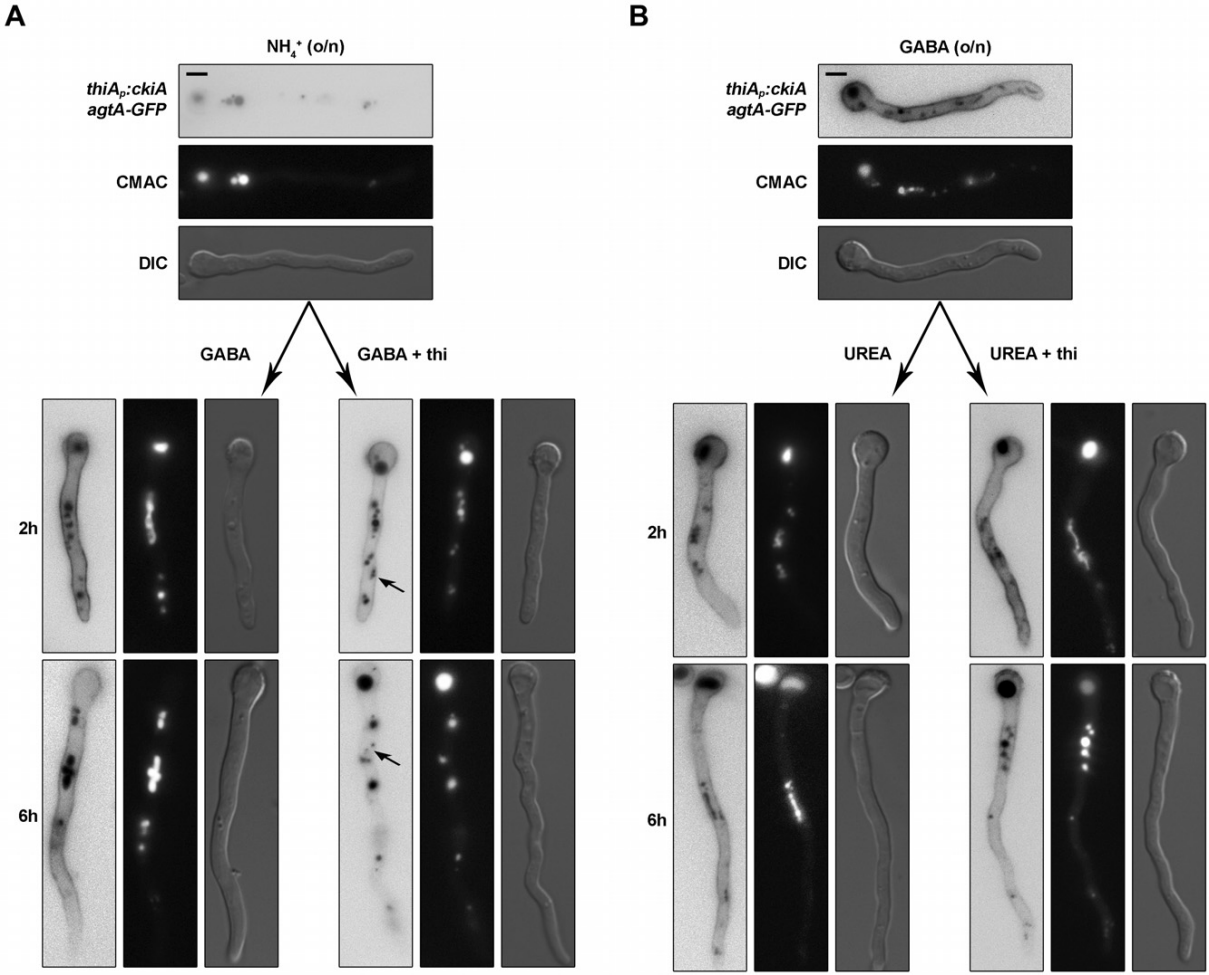
Localization of AgtA–GFP in a *ckiA* conditional mutant. Strain VIE050 was grown for 14–16 h in the presence of ammonium (NH4^+^ o/n) or GABA (GABA o/n) as nitrogen sources and then shifted to supplemented MM containing GABA or Urea as nitrogen sources, with or without 10 µM thiamine (+ thi) for the additional time indicated. For details on growth conditions, see *Experimental procedures*. Arrows indicate structures that are not stained by CMAC. Scale Bars: 5 µm.

We then investigated whether depletion of CkiA results in vacuolar miss-routing of the AgtA transporter. In *thiA^p^–ckiA* cells shifted to GABA (which leads to *agtA* expression), the absence of thiamine (i.e. CkiA-sufficient conditions) leads to plasma membrane (and secondarily in the vacuolar lumen) localization of AgtA–GFP, as in the wild type (Fig. 9A transfer to GABA, compare with Fig. 7, top panel). In contrast, if cells were shifted to GABA in the presence of thiamine (which results in CkiA downregulation), virtually all GFP fluorescence appeared into the vacuolar lumen after 6 h (Fig. 9A, transfer to GABA + thiamine). The vacuolar luminal localization of GFP (attached to the cytosolic C-terminus of AgtA) demonstrates that the transporter is sorted into the MVB pathway, but does not address whether the vacuolar GFP accumulation reflects endocytic or biosynthetic traffic (or both) of AgtA to the vacuole. Thus we carried out a second set of experiments in which we investigated the fate of AgtA in *thiA^p^–ckiA* cells pre-cultured on GABA that were transferred to medium containing urea, with or without thiamine. Urea represses strongly (albeit not completely, see below) *agtA* transcription but, at variance with ammonium, does not promote the endocytic internalization of the transporter (Apostolaki *et al*., 2009). Under these conditions (i.e. AgtA–GFP synthesis shut-off with or without CkiA downregulation), AgtA–GFP can still be seen in the membrane even after 6 h in the presence of thiamine (CkiA-downregulated), strongly suggesting the AgtA molecules that are already in the membrane are not internalized towards the vacuoles as a consequence of CkiA depletion. Repression of *agtA* transcription under identical conditions of those used for the experiment of Fig. 9 is shown in Fig. S4. Therefore these experiments suggest that the strong vacuolar GFP fluorescence seen in cells cultured on GABA following downregulation of CkiA largely results from direct (i.e. without passing through the plasma membrane) biosynthetic traffic of AgtA to the vacuoles.

### The *ckiA* alleles and CkiA depletion both result in proteolysis of the AgtA transporter

**Fig 10:**
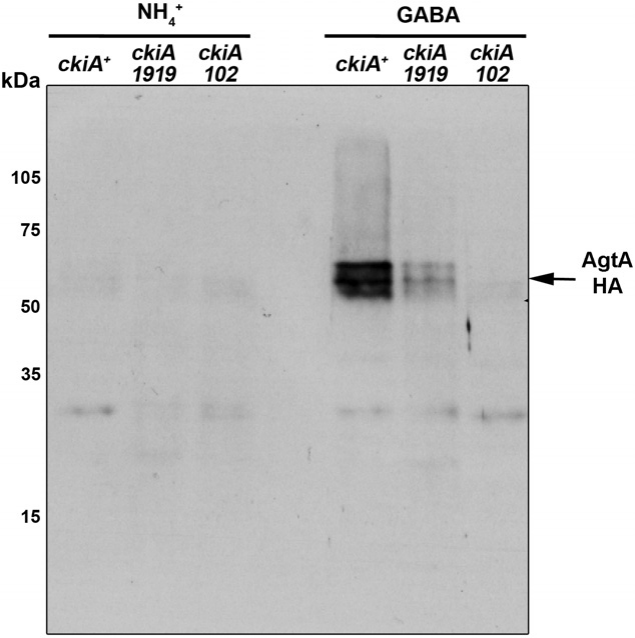
AgtA-3HA protein levels in *ckiA* mutants. Mycelia of LH121 (*ckiA^+^*), LH127 (*ckiA1919*), CAM13 (*ckiA102*) were pre-grown for 14 h at 30°C on ammonium as sole nitrogen source and transferred to either ammonium (NH_4_^+^) or γ-aminobutyric acid (GABA) for an additional 3 h incubation at 37°C. The *ckiA* allele used is indicated above each track. Loading was monitored by Coomassie blue staining and membranes were reacted with anti-HA primary antibodies and stained as described in *Experimental procedures*. Northern blots (not shown) carried out in parallel showed an identical pattern of de-repression of *agtA* after transfer to GABA (Apostolaki *et al*., 2009) for wild type and mutant strains. In a separate analogous experiment LH121 (*ckiA^+^*) was compared with AMC264 (*ckiA2*). The latter behaved exactly as *ckiA102* (not shown).

Delivery of AgtA to the vacuole should result in its proteolytic degradation. We thus investigated, using Western blots, whether *ckiA* mutations also result in degradation of an AgtA–(HA)3 fusion protein expressed from a gene replaced allele (Apostolaki *et al*., 2009). In strains carrying *ckiA2* or *ckiA102* the AgtA–(HA)3 steady state levels were markedly reduced, implying that the protein is degraded in the vacuole and that the fluorescence seen in the vacuole is mostly free GFP which is notoriously resistant to degradation (see below). In strains carrying the *ckiA1919* allele the signal is intermediate between that seen in the *ckiA^+^* strain and that seen in strains carrying *ckiA2* and *ckiA102*, in line with the less extreme phenotype of this allele (Fig. 10; not shown for *ckiA2*).

**Fig 11:**
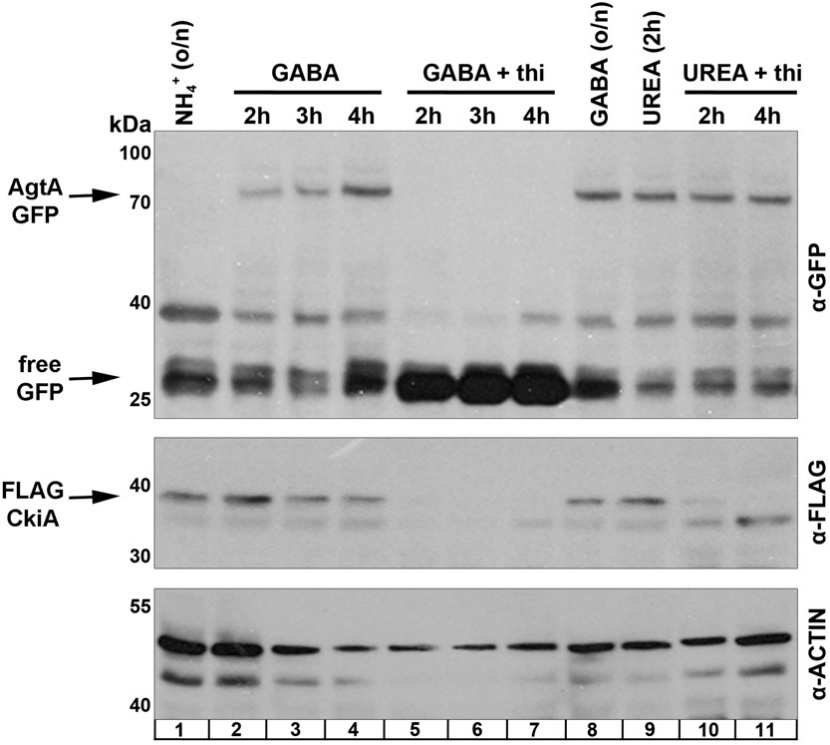
Degradation of AgtA–GFP after CkiA depletion. Strain VIE051, which carries *agtA–gfp* driven by its own promoter and *ckiA*-*flag*, driven by the *thiA* promoter was grown for 14 h at 30°C before transfer and/or addition of thiamine. For the eight tracks to the left of the panel, mycelia were grown for 14 h on ammonium (NH4^+^ o/n) and transferred to either GABA or GABA + 10 µM thiamine and grown for the time indicated. For the four tracks to the right of the panel mycelia was pre-grown for 14 h on GABA (GABA o/n) and then shifted to urea or urea + 10 µM thiamine for the time indicated. AgtA and its degradation products were monitored with an anti-GFP antibody, CkiA with an anti-FLAG antibody and total loading by an anti-actin antibody. For details see *Experimental procedures*.

We next used *thiA^p^–ckiA* strains expressing N-terminally FLAG-tagged CkiA kinase and AgtA–GFP, to monitor simultaneously the depletion of CkiA and the eventual degradation of the transporter. When AgtA–GFP synthesis was derepressed by shifting cells to GABA, if the medium lacked thiamine, both full-length AgtA–GFP fusion protein and GFP-containing degradation products were detected, in agreement with microscopy data (Fig. 11, lanes 2–4). However, in the presence of thiamine (CkiA-downregulated), only degradation products, of which the most conspicuous is free GFP, were seen (Fig. 11, lanes 5–7). On the other hand, when *agtA–gfp* was pre-derepressed for a period of time before adding both thiamine (to downregulate CkiA) and urea (to stop *agtA* transcription), full-length AgtA–GFP was clearly visible (Fig. 11, lanes 10 and 11), in complete agreement with the localization experiments in which plasma membrane-resident AgtA was unaffected by CkiA depletion. Thus these data strongly suggest that CkiA plays a role in a sorting step that precedes the delivery of AgtA to the plasma membrane.

## Discussion

### YAT localization and/or function are affected by mutations in numerous *A. nidulans* genes

Both recessive *ckiA* mutations and depletion (as a result of thiamine-mediated repression) of the CkiA protein result in defective utilization of a number of amino acids. This work strongly indicates that the partial CkiA deficiency resulting from any of the above mutant conditions affects a common mechanism specifically regulating intracellular trafficking of YAT transporters, leading to their miss-routing to the vacuole and subsequent degradation. *ckiA* mutants are able to grow on GABA, Gln and Asn, an ability shared by all the 22 non-*ckiA* mutants. The apparent YAT specificity of CkiA might explain why the utilization of GABA as a nitrogen source is unaffected by the mutations, as the GABA permease does not belong to the YAT family (Hutchings *et al*., 1999). Less straightforward is the interpretation of the finding that Gln and Asn utilization is also unaffected, as in *S. cerevisiæ* Asn and Gln are taken up by YAT transporters (Schreve *et al*., 1998; Regenberg *et al*., 1999). As the transporters responsible for their uptake in *A. nidulans* have not been identified, it is plausible that these belong to a different family.

Kinghorn and Pateman (1975) identified four loci where mutations result in inability to utilize glutamate. In three of these, *aauB*, *aauC* and *aauD*, mutations appear to affect a number of different amino acid transporters pleiotropically. As they map to chromosomes VII, II and VIII respectively, none of these loci can be *ckiA* (III) or *fbaA* (VI; Roumelioti *et al*., 2010). Sharma (1984) also identified phenotypically similar mutations mapping to chromosome VIII, whereas we have identified by transposon mutagenesis (Li Destri Nicosia *et al*., 2001) two insertional mutations mapping to chromosomes V and VII respectively, phenotypically very similar to *ckiA* mutations (Apostolaki, 2003). Thus, mutations in at least six (almost certainly more, see *Results*) loci affect amino acid uptake pleiotropically.

### Two mutations identified in this study have a highly specific effect on YAT localization

The growth phenotype of *ckiA1919* (Leu87Arg) is quite similar to that obtained when very low concentrations of thiamine (5–10 nM) are used to repress *thiA^p^–ckiA* partially, indicating that the efficient delivery of YAT transporters to the plasma membrane is extremely sensitive to *ckiA* deficiency. Leu87 is in the hydrophobic pocket that accommodates the purine ring of ATP (Fig. 3B). Arguably introduction of a positively charged, bulky arginine residue in this pocket might increase the *K_d_* for ATP and thus impair kinase activity. Such impairment is definitely mild because *ckiA1919* affects YAT trafficking without affecting viability or general growth rate.

A null *ckiA* mutation is lethal. Thus the two more extreme classical mutations can be more readily interpreted as loss-of-function mutations specifically affecting YAT trafficking. These mutations do not (*ckiA2*) or only slightly (*ckiA102*) affect colony morphology or growth on nitrogen or carbon sources other than amino acids. The inability of the cognate mutants to utilize amino acids resembles that seen in *thiA^p^–ckiA* strains at thiamine concentrations that, in contrast with *ckiA2* and *ckiA102*, also result in severely restricted growth on ammonium or on other utilizable nitrogen sources. Further evidence that *ckiA2* and *ckiA102* specifically affect YAT trafficking is their resistance phenotype to d-serine and *p*-flourophenylalanine. Again, full resistance to these toxic analogues is not accompanied by a strong growth defect, in contrast with the markedly restricted growth seen when CkiA is depleted. *ckiA2* (Glu37Lys) involves an N-terminal residue, universally conserved in casein kinases I of the δ/ε isotype, which lies in the surface of the modelled protein (Fig. 3C). Different surface residues of mouse CKIε mediate binding of its protein substrates (Dahlberg *et al*., 2009). It is thus likely that Glu37 is important for protein–protein interactions requiring a negatively charged surface side chain. The effect of *ckiA102* (Val295Phe) is more difficult to predict, as it maps to a residue included in motif conserved in the Pezizomycotina, but not in other fungi. Thus it is tempting to speculate that in Pezizomycotina such region is involved in specifically regulating, directly or indirectly, the delivery of YAT transporters to the plasma membrane. This region is immediately downstream from a conserved non-structured loop (see Fig. 3), and thus in a position suitable to engage in protein–protein interactions. The partners of these interactions and thus candidate CkiA substrates are currently unknown. Preliminary experiments using λ phosphatase digestion and comparison of AgtA–(HA)3 (Apostolaki *et al*., 2009) electrophoretic mobilities in wild-type and *ckiA* mutant backgrounds failed to provide any indication that AgtA could be a direct substrate of CkiA. It may be relevant that casein 1-δ modulates the traffic of the α sub-unit of the human epithelium sodium channel, while it does not phosphorylate directly this protein (Yan *et al*., 2007).

### Involvement of the ascomycete casein kinases I in intracellular trafficking

Our results imply that partial deficiency of *ckiA* results in the inappropriate redirection of newly synthesized AgtA and PrnB (and by implication of other YATs of *A. nidulans*) to the vacuole under physiological conditions which should lead to their delivery to the plasma membrane. While palmitoylated Yck proteins of *S. cerevisiæ* are involved in the localization of membrane proteins (Decottignies *et al*., 1999; Hicke, 1999; Feng and Davis, 2000; Marchal *et al*., 2000; 2002; Gadura *et al*., 2006), non-palmitoylated casein kinases belonging to the *HRR25*/*hhp1*/*hhp2* clade (Figs 3 and 4), play roles in DNA repair (Dhillon and Hoekstra, 1994; Ho *et al*., 1997), ribosome biogenesis (Ray *et al*., 2008), calcineurin signalling (Kafadar *et al*., 2003), meiosis (Petronczki *et al*., 2006) and circadian rhythms (Görl *et al*., 2001; He *et al*., 2006; Fan *et al*., 2009 and references therein), all of which are unrelated to intracellular traffic.

However, a role of *S. cerevisiae* Hrr25p in the ER-Golgi interface was suggested by the finding that an *hrr25* partial loss-of-function mutation suppresses the thermo-sensitive phenotype of *sec12-4*, the latter mapping in the Sar1p guanine nucleotide exchange factor. The mutation (T176I; Fig. 3A) acts as a specific suppressor, as a null mutant does not suppress *sec12-4* (Murakami *et al*., 1999). In the modelled structure of Hrr25p, Thr176 is a surface residue located in the strictly conserved region that hinges the β-sheet and α-helix moieties (Fig. 3C). It is thus likely that this residue is involved in a protein/protein interaction. Two recent articles lend support to a role of Hrr25p in cell trafficking. Gao and Kaiser (2006) reported that Ltv1p is a component of an endosomal GTPase complex that is necessary for Gap1 delivery to the plasma membrane. While the involvement of Hrr25p was not addressed in that study, Ltv1p was shown to be an Hrr25p substrate in a different context (Schäfer *et al*., 2006). More importantly, Lord *et al*. (2011) established that Hrr25p localizes also to the Golgi and specifically phosphorylates Sec23p. It is proposed that this phosphorylation establishes the directionality of the ER-Golgi traffic and prevents the back fusion of the COPII vesicles with the ER.

While these data point out to a previously unsuspected role for casein kinases I-δ in cell trafficking in *S. cerevisiæ*, our work establishes that in *A. nidulans* a specific function of CkiA is necessary for the delivery of amino acid transporters to the plasma membrane, a finding that is unprecedented in the literature*.*

Under physiological conditions under which the uptake of their substrates is no longer needed, transporters *en route* to the plasma membrane can be diverted to the endosomal system (and thus to the vacuole) from the Golgi. The best-studied example is that of the general amino acid permease Gap1p, whose sorting to endosomes involves ubiquitination (Rubio-Texeira and Kaiser, 2006). CkiA activity could inhibit, directly or indirectly a similar sorting step, so that re-routing to endosomes would occur when its activity is defective. It is noteworthy than in *S. pombe*, the amino acid permease Aat1p is retained in the Golgi in conditions of nitrogen sufficiency, and sorted to the plasma membrane in conditions of nitrogen starvation (Nakase *et al*., 2012) which illustrates the possible variations in intracellular traffic within the ascomycetes.

The fact that Hrr25p plays a role in determining the directionality of COPII-mediated ER-to-Golgi transport through phosphorylation of the coat component Sec23p (Lord *et al*., 2011) would also be consistent with the possibility that, when CkiA activity is defective, transporters incorporated into ER-derived vesicles are delivered to the vacuoles without passing through the Golgi. For example, these transporters could use the autophagy pathway, as the ER provides membranes for the biogenesis of autophagosomes (Mizushima *et al*., 2011). YAT transporters would next gain access to the vacuolar lumen after fusion of autophagosomes with vacuoles. If this were the case and assuming at least a partial conservation of function of the CkiA/Hrr25p orthologues, one implication of our studies is that Hrr25p could have a role involving anterograde trafficking of YAT transporters across the ER-Golgi interphase, a role which has not been investigated. Thus, it would be most interesting to know the fate of a secretory cargo, dependent on COPII-mediated ER-Golgi transport (such as Gap1p Malkus *et al*., 2002), in a yeast strain inactivated or depleted for Hrr25p, as well as to investigate whether CkiA is able to phosphorylate *A. nidulans* Sec23 (Pantazopoulou and Peñalva, 2009).

## Experimental procedures

### Media growth conditions and genetic methodology

Minimal (MM) and complete (CM) media as well as growth conditions for *A. nidulans* were described by Cove (1966). Auxotrophies were supplemented at the concentrations given in http://www.fgsc.net/Aspergillus/gene_list/supplement.html, or as specifically detailed in *Supplementary Experimental procedures*. For growth tests, conidiospores were inoculated on minimal media supplemented with the appropriate nitrogen source and incubated at 37°C for 48 h unless otherwise indicated. Mutations were selected spontaneously or after chemical mutagenesis as described in *Supplementary Experimental procedures*. Crosses between *A. nidulans* strains and construction of diploids were described by Pontecorvo *et al*. (1953). Haploidization was as described by Hastie (1970).

### Strains, plasmids and genomic libraries

Auxotrophic and morphological mutations of *A. nidulans* strains are compiled by A.J. Clutterbuck (http://www.gla.ac.uk/acad/ibls/molgen/aspergillus/index.html), where gene symbols are described. The strains used and constructed during this work are listed in Table 1. All strains are *veA1*.

### *Escherichia coli* strains

The *E. coli* strains used were JM109b and DH10B.

### Vectors and plasmids

Those are listed in *Supplementary Experimental procedures*, Tables S1–S3.

### Transformation methods

Transformation of *E. coli* was carried out as described by Sambrook and Rusell (2001) and Dower *et al*. (1988). Transformation of *A. nidulans* is described by Tilburn *et al*. (1983).

### DNA manipulations

Plasmid and cosmid preparation from *E. coli* strains was carried out as described by Sambrook and Rusell (2001) or by using the Qiagen Plasmid Mini and Plasmid Midi kit according to the manufacturer's instructions. Cosmid pool preparation is described in the *Supplementary Experimental procedures*. DNA digestion and cloning strategies were carried out as described by Sambrook and Rusell (2001). Genomic DNA extraction from *A. nidulans* is described by Lockington *et al*. (1985). Southern blot analysis was carried out according to Sambrook and Rusell (2001), and details of specific procedures are given in the *Supplementary Experimental procedures*. High-fidelity PCR reactions were carried out using the kit Expand™ Long Template PCR System (Roche). Conventional PCR reactions were carried out using Taq polymerase (Promega) and the REDTaq® ReadyMix™ (Sigma Aldrich). TA cloning was carried out using the pGEM™-T easy vector system (Promega). DNA bands were purified from agarose gels using the Wizard PCR preps DNA purification system (Promega) and the MinElute Gel Extraction Kit (Qiagen). Cloning and amplification of the replacement cassettes were carried out using Phusion® Flash High-Fidelity PCR Master Mix (New England Biolabs). The ^32^Pα-dCTP labelled DNA molecules, which were used as gene-specific probes, were prepared using the Megaprime™ DNA labelling systems kit (Amersham LIFE SCIENCE) or the Random Hexanucleotide Primer kit and purified on MicroSpin™ S-200 HR columns, following the supplier's instructions (Roche Applied Science). DNA sequences were determined in MWG AG Biotech and GENOME Express. The primers used are detailed in Table S4.

### *ckiA* cloning

This was carried out by complementing appropriate *ckiA102* strains with the minimal ordered compressed cosmid library of the third chromosome of *A. nidulans* constructed in the pWE15 and pLORIST2 cosmids (Prade *et al*., 199*7*) or the AMA-NotI genomic library constructed in the self-replicating pRG3NotI plasmid (Osherov and May, 2000), which was re-amplified as described in Apostolaki (2003). Further details are given in the *Supplementary Experimental procedures*.

### Construction of *ckiA* deletions

These are detailed in *Supplementary Experimental procedures*.

### Construction of strains containing in-locus *ckiA* transcriptional and translational fusions

A cassette containing ckiA::sgfp::AFpyrG was used to transform TNO2A25. Three cassettes containing thiA^p^::ckiA, thiaA^p^::FLAG–ckiA and AFriboB–thiAp::gfp::ckiA fusions were constructed. The first two were used to transform the AMC314 strain. Selection of transformants with fully restored ckiA function was carried out on MM with glutamate as sole nitrogen source. The third cassette was used to transform TNO2A7 and selection was carried out on MM media with urea as sole nitrogen source, in the absence of riboflavin. The intact single copy in-locus replacements were confirmed by Southern blot analysis. Further details are given in *Supplementary Experimental procedures*.

### RNA manipulations

Total RNA extraction from *A*. *nidulans* was carried out using the RNAPLUS™ (Q-BIOgene) or TRIzol® Reagent (Invitrogen) according to the corresponding manufacturer's instructions and also as described by Chomczynski and Sacchi (1987) and Lockington *et al*. (1987). RNA was separated on glyoxal agarose gels as described by Sambrook and Rusell (2001). The hybridization technique was described by Church and Gilbert (1984). To monitor RNA loading, the γ-actin gene of *A. nidulans* and the radish *18S rRNA* gene were used as probes.

### Protein manipulations

Both total proteins and membrane-bound proteins were analysed in Western blots as described respectively by Vangelatos *et al*. (2010) and Calcagno-Pizarelli *et al*. (2007). Further details are given in *Supplementary Experimental procedures*.

### Amino acid uptake assays

^3^H-labeled amino acid uptake was measured in germinating conidia and was carried out following the procedures of Robinson *et al*. (1973) and Tazebay *et al*. (1995). Details are given in the *Supplementary Experimental procedures*.

### Fluorescence microscopy

Germlings of strains AMC132, CAM51, AMC314 and AMC129 were grown for 16 h at 25°C on supplemented watch medium (WMM) containing GABA as sole nitrogen source (Peñalva, 2005). *agtA* expression was modulated as described in Apostolaki *et al*. (2009). Germlings of strains TpA4, VIE047, VIE050 and VIE172 were obtained after growth on supplemented MM at 25°C for 14–16 h in the presence of ammonium or GABA as nitrogen sources and then shifted to supplemented MM containing thiamine to a final concentration of 10 µM and GABA or urea as nitrogen sources and incubated for additional 2–6 h. Details of image acquisition are described in *Supplementary Experimental procedures*.

### Bioinformatic tools and databases

See *Supplementary Experimental procedures*.
