# Supplementary material for: Aspergillus nidulans CkiA is an essential casein kinase I required for delivery of amino acid transporters to the plasma membrane
Source: Mol Microbiol. 2012 Apr 10;84(3):530–49. doi: 10.1111/j.1365-2958.2012.08042.x (PMC3491690; doi:10.1111/j.1365-2958.2012.08042.x)
Supplement: Supplementary file 1 [file mmi0084-0530-SD1.pdf]

Supporting Information

Aspergillus nidulans CkiA is an essential casein kinase I required for delivery of amino acid transporters to the plasma membrane

Angeliki Apostolaki*[1§], Laura Harispe*[1,2†], Ana María Calcagno-Pizzarelli[3], Ioannis Vangelatos[4], Vicky Sophianopoulou[4], Herbert N. Arst Jr[3,5], Miguel Angel Peñalva[2], Sotiris Amillis[6#] and Claudio Scazzocchio[1,3#]

Supplementary Figures

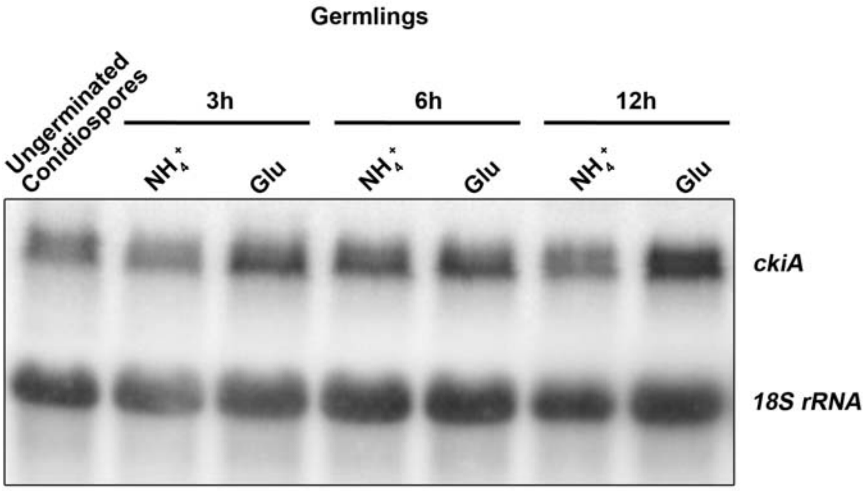

Fig. S1: Identification of the *ckiA* transcript.

Northern blot of RNA extracted from resting conidiospores and germlings grown at 37° C on MM with the either ammonium or glutamate as nitrogen source for the time indicated.

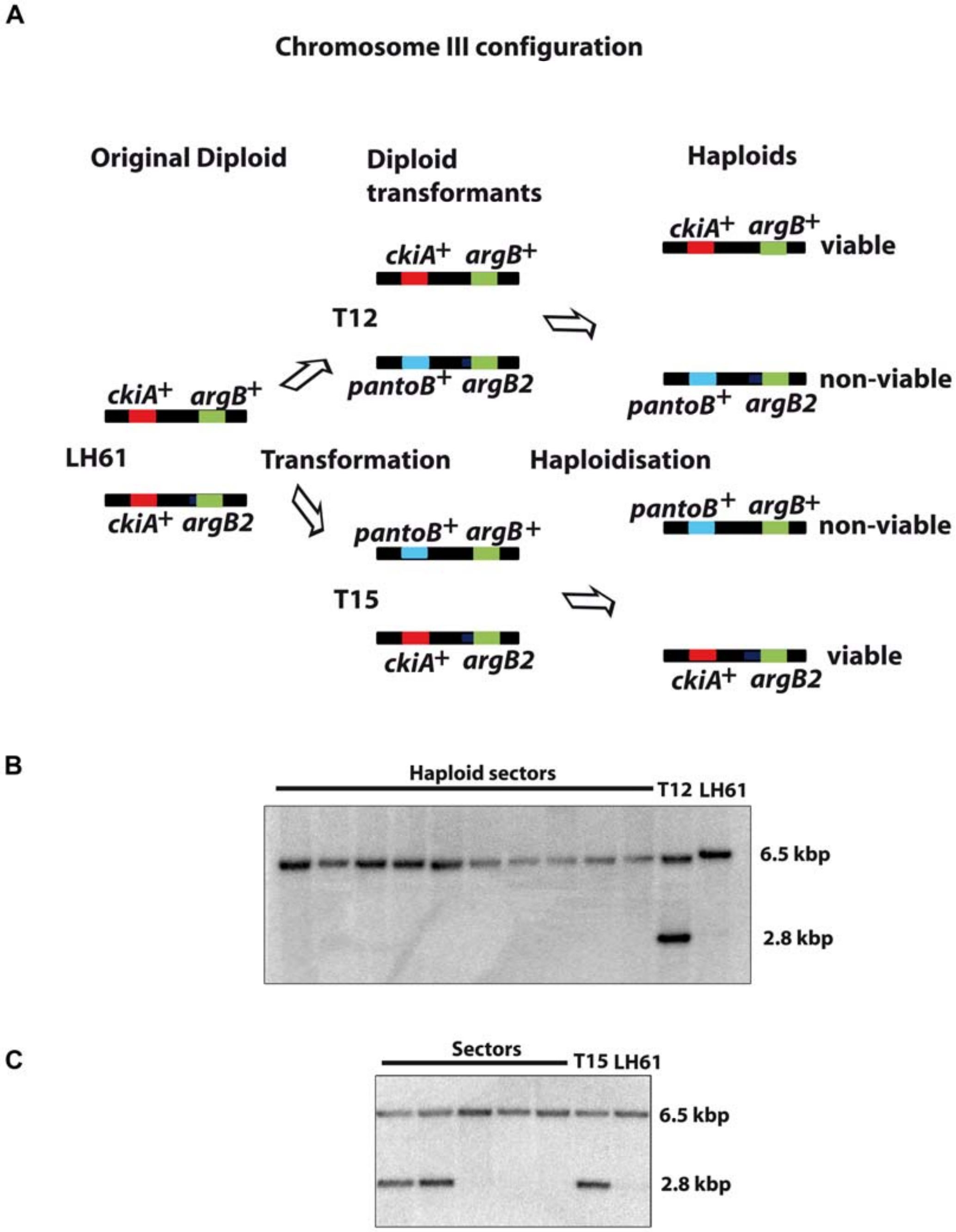

Fig. S2: *ckiA* is an essential gene: inactivation in a diploid strain.

Panel A shows a scheme of the chromosome III homologues of strain LH61. In red *ckiA*, in green *argB*. The strain is $ckiA^{+}/ckiA^{+}$ homozygous and $argB^{+}/argB2$ heterozygous. It is also homozygous for *pantoB100* in chromosome VII and thus a pantothenate auxotroph. If a *ckiA* deletion is a recessive lethal, as indicated by the heterocaryon experiments, the $pantoB^{+}$ gene (in blue) could substitute one of the *ckiA* genes, resulting in a *ckiAΔ* hemizygote which is $pantoB^{+}$ (shown in blue). This can occur in coupling (as it occurred in T12.) or in repulsion with *argB2* (as it occurred in T15). Panel B, Sothern blot of

representative sectors originating form the T15 diploid transformant. (*XbaI* digest) showing the 6.5 kB band of *ckiA* present in the recipient strain; and in the diploid transformant, the two bands of 6.5 kB and 2.8 kB representing the intact *ckiA* gene and the restriction fragment predicted to result from the *pantoB*$^{+}$ substitution, respectively. All haploid (*argB*$^{+}$) strains show only the wt *ckiA* restriction fragment. Panel C, representative sectors originating from the T15 diploid, analysed as above. Three haploid strains (*argB2*) behave as predicted, while the two putative haploid sectors (to the left of the panel), which show both bands were shown on further testing to be prototrophic for all markers and actually diploid. A 1140 bp PCR fragment corresponding to the region downstream the deletion cassette (from position +1392 to position +2530 relative to the *ckiA* translation start) was used as a probe.

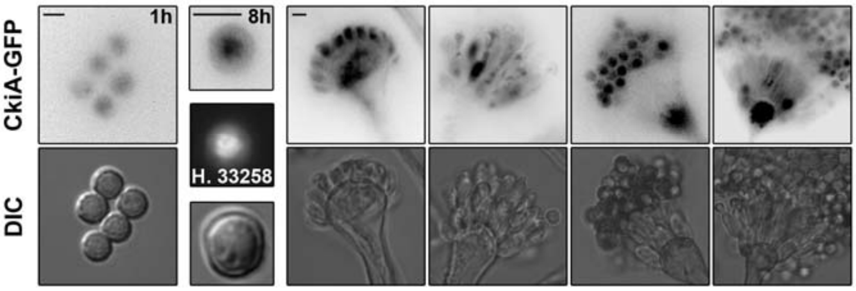

Fig. S3: Localisation of CkiA in conidia incubated in liquid MM for the time indicated and in condiophores grown on agar blocks, on urea as sole nitrogen source. Scale Bars: 5 µm. Intact conidiophores show a quite heterogeneous distribution of CkiA-GFP, which may be correlated with different developmental stages. Mature conidia always show the presence of CkiA-GFP. Noticeably, at least in some mature conidiophores the protein is present in conidia and in the vesicle, but absent from metullae and phialides.

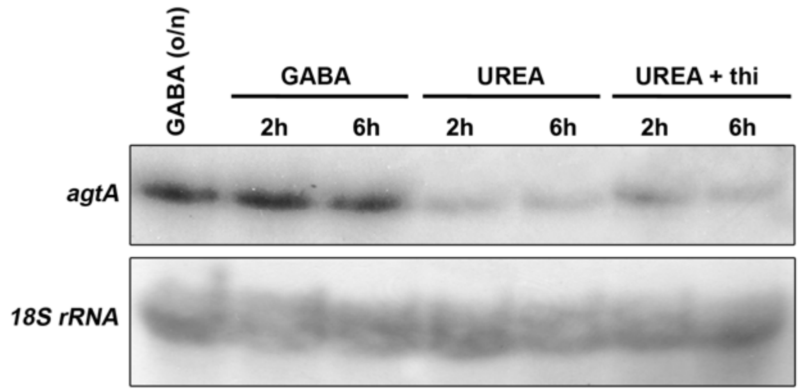

Fig. S4. Northern blot analysis of strain VIE051, which carries *agtA*-gfp driven by its own promoter and *ckiA*-flag, driven by the *thiA* promoter, grown for 14 h at 30° C on GABA (o/n) before transfer to urea or urea with addition of 10 μM thiamine (thi) for the time indicated.

Supplementary Experimental procedures

Supplements: Ammonium L **(**+**)-**tartrate, and urea were used at 5 mM, acetamide, nitrate ($NaNO_3$), and nitrite ($NaNO_2$) at 10 mM. Uric acid, xanthine, hypoxanthine, adenine and allantoin at 0.1 mg/ml, as sole nitrogen sources. Amino acids were generally used at 5 mM as carbon or nitrogen sources in solid media (unless differently described in the text) and at 10 mM as nitrogen sources in liquid media. Aspartate and glutamate were used as monosodium salts (SIGMA). In MM, glucose was used as a carbon source at 1 % w/v. Acetate was used at 50 mM as a carbon source. The amino acid toxic analogues D-serine and DL-parafluorophenylalanine (FPA) were used at 5 mM and 55 μM respectively. Uracil and uridine were used at 5 mM and 10 mM respectively.

ckiA$^-$ *mutant selection and characterisation*: *ckiA2* was selected after N-methyl-N'-nitro-N-nitrosoguanidine mutagenesis of a strain of genotype *biA1;puA2;fwA1* as a faster growing, nitrogen-starved sector (Herman and Clutterbuck, 1966) on biotin-supplemented glucose minimal medium containing limiting (10 nM) putrescine and 5 mM delta-aminovaleric acid as nitrogen source. *ckiA102* was isolated as described by Arst *et al.* (1981) as a spontaneous mutation conferring resistance to 50 mM proline to a strain carrying *proA6* (conferring proline auxotrophy) and *sasA60* (conferring toxicity to

proline) (Sharma, 1984). The selection of novel mutants resistant to D-serine or FPA (including *ckiA1919*) was carried out during this work by inoculating conidia of CS2290 on solid supplemented MM medium containing D-serine with uric acid as a sole nitrogen source. After 4-5 days of incubation at 37° C, mutant sectors were recovered and purified on supplemented MM containing ammonium as a nitrogen source. Putative *ckiA fbaA1013* double mutants were checked by outcrossing to a wild type, with recovery of both parental classes and for the strains tested in Fig. 1B, also by confirming the presence of the relevant *ckiA* mutation after PCR amplification of the *ckiA* ORF (see text for the nature of each mutation).

Plasmids

Table S1. Vectors

Vector	Provider
**pBluescript KS+**	Stratagene
**pBluescript SK+**	Stratagene
**pGEM®-TEasyVector**	Promega

Table S2. Previously published plasmids

Plasmid	Insert	Provider
**pSF5**	γ-actin gene of *A. nidulans*	Fidel *et al.*, 1988
**pRG3**	18S ribosomal RNA gene of radish	Delcasso-Tremousaygue *et al.*, 1988
**pRG3NotI**	AMA-*NotI* genomic library	Osherov and May, 2000
**pAB1**	a 1649bp PCR product of the *agtA* gene	Apostolaki *et al.*, 2009
**pnirAΔ**	*riboB* gene of *Aspergillus fumigatus*	Schinko *et al.*, 2010
**pERE-nirA^{c}1-GFP**	*gfp* gene (sGFP-TYG version)	Bernreiter *et al.*, 2007
**p1439**	a translational fusion between a 5 Gly-Ala (5GA) linker and the *sgfp* followed by the *A. fumigatus pyrG* gene	Yang *et al.*, 2004

Table S3. Plasmids isolated or constructed in this work

Plasmid	Vector	Insert
**pRG3C**	pRG3NotI	a 4201 bp fragment corresponding to the sequence between positions 285224-289425 of contig 1.78
**pRG3B**	pRG3NotI	a approximately 14 kb fragment that completely includes the insert of plasmid pRG3C
**pAA21**<br>**pAA22**	pKS+	the *SmaI-NotI* fragment of the insert (4203bp) contained in *pRG3C* cloned in the *NotI* site of *pKS+*
**pAA51**<br>**pAA55**<br>**pAA58**	pGEM®-T EasyVector	a 6143 bp PCR product obtained using L12G07 cosmid as template and primers C10-C11 (contig 1.78, 285431-291574)
**pAA61**<br>**pAA62**<br>**pAA63**	pKS+	the 3099 bp *Sal*I-*BglII* fragment of the insert contained in *pAA51* cloned in the *EcoRV* site of *pKS+* (contig 1.78, 287061-290160)
**pT^{P}CkiA**	pGEM®-T EasyVector	the *thiA^{p}::ckiA* cassette
**pT^{P}FLAG-CkiA**	pGEM®-T EasyVector	the *thiaA^{p}::FLAG-ckiA* cassette
**pT^{P}GFP-CkiA**	pGEM®-T EasyVector	the *AFriboB-thiAp::gfp::ckiA* cassette
**pCkiAKO**	pGEM®-T EasyVector	the 5'UTR-*AFriboB*-3'UTR cassette

Table S4. List of primers used in this study

Name	Sequence 5' to 3'
**PRG3F**	GAATTCGAGCTCGGTACC
**PRG3L**	AAGCTTGCATGCGCGGCC
**C8**	GATAGTAGAGCGAATGACG
**C9**	AAGTACCAGAAGAACGCTGC
**C10**	GCAGTAGAGAGTACATACG
**C11**	TAAGCATGAGAACTCGCTGC
**DelK1**	CAGAGCAGAACCGACTAAGAGAACTGGAGC
**DelK2**	AGACAAGTCACAACAAAGAGAGGCTGAGCC
**DelK3**	CAAATCAGAAGGCTCAGCCTCTCTTTGTTGTGACTTGTCTAGAAGAGTCACGTCGGCTACTCGACTAACG
**DelK4**	CGGAACGCAGCCTAACGCCGAAGACTATGAGATTAGTCAGTAGCTTCCTTATTGCTGGAGCAGTGATGCG
**DelK5**	CTGACTAATCTCATAGTCTTCGGCGTTAGG
**DelK6**	GATGACTGGGATTGATCGACGTGTCTAAGG
**ABFW**	AGTACTAACAGCAACTACCGACTGAGTA
**ABRV**	AACACTGAGCTTTTGTGTGACAATACTT
**BCFW**	GACTCATTCTCAGTTGTACTACCTAC
**BCRV**	AATGTACAAGCAATCTTAACATCAGTAGTC
**CKPtoUF**	GTTTCTGAGAAGACTAGCTCTGAACTG
**CKPtoUR**	GTTTTCTTGTCATTATATGCTGTATCCTAC
**CKNF**	ACTAACCTGTGTAAAGGTACCAACA
**CKNR**	GAGTGTAGTTGAGGTAGATGGAGAA

**SouthFCk**	ATTATGAAGCCTAGCTTCCTGTTTT
**SouthRCk**	ACATCAATGAGGGTCAAAGTAATGT
**CkiA P1**	CGTAAGATCGGAAGCGGTAG
**CkiA P2**	CCATCAAGCTCGAAAGCGTC
**CkiA P3**	CAGCGCCTGCACCAGCTCCCATCCTGTCGCTCCCTCC
**CkiA P4**	GCATCAGTGCCTCCTCTCAGACTAGGTTCCGTGTAGGGAAC
**CkiA P5**	GAAGGAGCGCAAACAGTTGG
**CkiA P6**	GTCTCATCCACATAGGAATAGG
**CkiAp1439 F**	GGAGGGAGCGACAGGATGGGAGCTGGTGCAGGCGCTG
**CkiAp1439 R**	GTTCCCTACACGGAACCTAGTCTGAGAGGAGGCACTGATGC
**CkiA 5’ ApaI F**	CGGGGGCCCCTTTGTTCGAATTGACGATGCCCTG
**CkiA 5’ SphI R**	CGGGCATGCGCTTTGATACCTACGCCCCCAACAC
**CkiA 3’ SphI F**	CGCGGCATGCGTATCTAAAACCTAGGTTCCGTGTAG
**CkiA 3’ NdeI R**	CGCGCATATGCACAGCCACGGCTCTCTCAGAGCC
**ThiA^p SphI F**	CGGGCATGCCGACCTGGCACCTACAGAAGAATCC
**ThiA^p EcoRI R**	CGGGAATTCGTTGACTCAGTTCAATGGTTCGACTATAG
**CkiA EcoRI F**	CGGGAATTCATGACGACCATGGTGAGTTTTTTCCCC
**CkiA NdeI R**	CCGGCATATGGGGGGTCGTCATCTTTTTCTCCATAATAC
**AFriboB SphI F**	CCCGGGCATGCAAGCTTGATATCACAATCAGC
**AFriboB SphI R**	CCCGGGCATGCCCCGGGCTGCAGGAATTCGATAAG
**GFP EcoRI F**	CCGGGAATTCATGGTGAGCAAGGGCGAGGAGC
**GFP EcoRI R**	CCGGGAATTCCTTGTACAGCTCGTCCATG
**thiA^{p}FLAG EcoRI R**	CGCGGAATTCCTTGTCATCGTCGTCCTTGTAGTCCATGTTGACTCAGTTCAATGGTTCGACTATAG

Southern blot analyses: The cross hybridisation of pRG3C to cosmid L12G07 was tested in *HindIII* and *EcoRI* digests with the purified *SmaI-NotI* fragment of plasmid pRG3C. The restriction enzymes used to monitor the copy number of *ckiA* in the genome of CS2498, CS1901 and CS1902 were *NcoI*, which does not have a recognition site within the genome sequence included in *ckiA* probe employed (a PCR product using primers pRG3F and prG3R and plasmid pRG3C as a template) and *EcoRI* that cuts twice within this sequence. Southern blot analysis of the transformants obtained with pAA21 and pAA22 plasmids was carried out using *HindIII* and the latter PCR product as a probe. To characterise the transformants obtained with pAA61, pAA62 and pAA63 (see below), a Southern blot was carried out using *BglII* and the purified *BglII-SalI* fragment from plasmid pAA51 as a probe.

Identification of ckiA using the AMA-NotI genomic library: this gene library was constructed in the self-replicating plasmid pRG3-*Not1* (Osherov and May, 2000) carrying the *pyr4* gene of *Neurospora crassa* as a selection marker, which complements *pyrG89* mutation of *A. nidulans*. CS1901 carrying *ckiA102* and *pyrG89* (resulting in a uracil/uridine requirement) was transformed with 10 μg of the amplified library. Selection of transformants was carried out on supplemented MM containing 10 mM glycine as a sole nitrogen source in the absence of uracil and uridine. The transformants were tested for their ability to grow on a number of amino acids (proline, tryptophane, arginine, glutamate, β-alanine, leucine and serine) as sole nitrogen sources and by their sensitivity to the toxic analogues D-serine and FPA (on uric acid as a nitrogen source). The free plasmids harboured by nineteen independently obtained transformants, which were sensitive to both toxic analogues and able to grow on amino acids as nitrogen sources, were recovered by electroporating the *E. coli* strain DH10B with dialysed genomic DNA (extracted from mycelia grown in the presence of glycine as nitrogen source). The recovered plasmids were characterized by restriction (using *BamHI*, *SphI* and *KpnI* that cut once in pRG3-*NotI* polylinker) and one representative from each restriction profile was used to re-transform CS1901. Two plasmids, pRG3B and pRG3C, were able to fully complement *ckiA102*. Sequencing showed that pARG3C carries a 4301 bp insert comprising the whole putative open reading frame of ANID_04563.1 (accession number of the last release of the Broad Institute database, http://www.fgsc.net/aspergenome.htm), together with 322 bp of its upstream region and a partial sequence of ANID_04562.1. pARG3B carries an approximately 14 kb insert, which fully comprises the insert of plasmid pARG3C. In order to check whether the whole insert carried in plasmid pARG3C was able to complement *ckiA102* after single ectopic integration, the latter was cloned in the integrative vector pBKS+ to give plasmid pAA2 (Table S3). pAA2 was used to transform CS1901 in the same conditions as previously mentioned. The insert carried by plasmid pARG3C only affords full complementation of the *ckiA102* mutation when present in multiple copies, but only complements partially *ckiA102* by single copy ectopic insertion at a number of different positions in the genome when cloned in the integrative vector pAA2. A full wild type phenotype was obtained only after single *in locus* insertion

of the pAA2 plasmid (resulting presumably in correction of the mutation), which was visualized by Southern blot analysis as a locus duplication.

Identification of ckiA using the cosmid library: the 190 cosmids of the minimal ordered compressed cosmid library of the third chromosome of *A. nidulans* were divided into 8 pools. For cosmid-pool preparation, the corresponding bacterial colonies were inoculated on solid LB medium. Colonies were harvested after two days of growth at 37º C, resuspended in 100 ml liquid LB medium and incubated for 1 h at 37º C under agitation (200 rpm). Cosmid DNA was then extracted using the Qiagen Plasmid Midi kit according to the manufacturer's instructions. CS1901 was transformed with 10 μg of each pool. Eventually, only one cosmid L12G07 was able to complement the mutation. L12G07 showed cross-hybridisation with the insert contained in pRG3C (see above). Selection of transformants was carried out on supplemented MM containing 10 mM glycine as a sole nitrogen source. In order to isolate the whole *ckiA* gene in an integrative vector, first pAA5 was constructed (Table S3) by ligating the integrative pGEM^{TM}-T easy vector to a 6.1 kb insert, which was PCR amplified using cosmid L12G07 as a template. This was 1918bp longer than the pRG3C insert upstream the ANID_04563.1 gene sequences and shorter by 207 bp in the adjacent ANID_04562.1 sequences. pAA5 was used to transform CS1901 in the same conditions as previously. Subsequently, the *BglII-SalI* fragment (3099 bp) of pAA5 insert was subcloned into pBKS+ to construct plasmid pAA6 (Table S3), which was also used to transform CS1901 as previously. This fragment contains the complete ANID_04563.1 extended by 1053 bp upstream and by 595 bp downstream, and is able to complement *ckiA102* in a single copy, as differently from pARG3C and derived plasmids (see above) it presumably carries a complete 5' upstream region.

***Construction of ckiA deletions*:** *ckiA* deletion was attempted, first, by replacing the whole *ckiA* ORF (from position -192 bp with respect to the translation initiation codon up to 110 bp after the translation termination codon) employing a method based on the concurrent *in vivo* recombination of two overlapping DNA fragments *in locus*, each carrying a partial sequence of the deletion cassette, resulting in replacement of the corresponding native sequence by a reconstructed marker gene. Each DNA fragment contained the

upstream or the downstream region flanking the deleted part of *ckiA*, a part of the marker gene unique to each fragment and a common (overlapping) region of the marker gene which would promote recombination between the two partial sequences of the deletion cassette. These fragments were constructed by the DJ-PCR method (Yu *et al*., 2004). The regions upstream (from position 289.013 to position 292.082 of contig 1.78) and downstream (from position 289.013 to position 292.082 of contig 1.78) the *ckiA* ORF, to be called respectively region A and C, were amplified using the primer pair Delk1-Delk2 (for region A) and Delk5-Delk6 (for region C) and genomic DNA from strain CS2498 as template. The *pantoB* marker gene, to be called region B, was amplified from genomic DNA of CS2498 using the primer pair Delk3-Delk4. Regions A and C were independently fused by DJ-PCR with region B. Then, a nested fragment of each of these fused DNA segments was amplified using the primer sets ABFW-ABRV (for the region A+B) and BCFW-BCRV (for the region B+C), giving fragments D and E respectively. Fragments D and E contain a region of homology of approximately 1,5 Kb with the upstream and downstream regions of *ckiA* gene respectively as well as the upstream or downstream incomplete portion of the *pantoB* gene, which alone are not capable of complementing *pantoB100* mutation. Furthermore, D and E overlap to each other by approximately 1Kb, in the region encoding the ORF of *pantoB*. Thus, a triple homologous recombination event is required to obtain transformants prototrophic for pantothenic acid. This construction was used to transform haploid strain LH59 and diploid strain LH61. Transformants were selected in the absence of pantothenate. Diploid transformants, carrying integrations in *ckiA,* were identified as heterozygous by PCR using the primer pair CKPtoUF (hybridizes with *pantoB* gene)-CKPtoUR (hybridizes with the region downstream the E fragment) and genomic DNA as template. For five selected transformants, *in locus* integration was also confirmed by Southern blot analysis using the enzyme *XbaI* that cuts only once in the *pantoB* gene (and none in the deleted *ckiA* sequence) and hybridizing with a purified 1140 bp PCR fragment corresponding to the region downstream of the deletion cassette (from position +1392 to position +2530 with respect to the *ckiA* translation start) as a probe. This probe was amplified from genomic DNA of CS2498 with the primer pair SouthFck-SouthRck. All of the

examined transformants carried a single gene replacement in only one of the two chromosome III homologues. Haploidisation of diploid transformants was carried out on CM in the presence of benlate at 5 mg/ml (Hastie, 1970). Southern blot analysis (as above) was used to check the status of the *ckiA* gene in the issuing haploids. Figure S2 illustrates the results obtained.

Additionally, a second deletion cassette containing the 5'UTR-*AFriboB*-3'UTR fusion was constructed by sequential cloning of the corresponding PCR-amplified fragments in the *ApaI*, *SphI*, and *NdeI* sites of the polylinker of the pGEM-T Easy vector (Promega) using primer pairs carrying restriction enzyme adaptors (Table S4). The *ckiA* 1256 bp upstream region (starting at 285961 of contig 1.78 and ending 670 bp before the translation initiation codon), and the *ckiA* 1261 bp downstream region (starting at 289074 of contig 1.78, immediately after the translation termination codon), were, first, amplified using as template genomic DNA of TNO2A7 and the primer pairs CkiA 5' *ApaI* F-CkiA 5' *SphI* R, CkiA 3'*SphI* F-CkiA 3'*NdeI* R respectively. Subsequently, they were digested and cloned into the pGEM-T Easy vector, resulting in plasmid pCkiAKO. The *AFriboB* amplified from plasmid p*nirA*Δ using the primer pair AFriboB *SphI* F-AFriboB *SphI* R was cloned at the *SphI* site of the above plasmid. The resulting transformation cassette was amplified by the primer pair CkiA 5' *ApaI* F-CkiA 3' *NdeI* R and used to complement the *riboB2* auxotrophic mutation of strain TNO2A7 by selecting on MM media with urea as sole nitrogen source, in the absence of riboflavin.

Construction of strains containing in-locus ckiA transcriptional and translational fusions: The cassette containing the *ckiA*::*sgfp::AFpyrG* sequences was constructed by joining three different PCR fragments, as described by Szewczyk *et al.* (2006). DNA fragments corresponding to the central part of the construction were amplified from the p1439 plasmid using the primer pair CkiAp1439 F-CkiAp1439 R (Table S4). The upstream flanking sequence of the *sgfp* ORF is a 1109 bp fragment (starting at 287993 of contig 1.78) followed by the 5GA linker, while the downstream flanking sequence is a fragment containing a 1239 bp fragment corresponding to the 3' end of the *ckiA* gene (starting at 289115 of contig 1.78), 2bp just after the chain termination codon. Both flanking fragments were amplified from genomic

DNA of a TNO2A7 strain using the CkiA P1-CkiAP3 and CkiA P4-CkiA P6 primer pairs respectively. The entire fusion cassette was amplified using the CkiA P2-CkiA P5 nested primer pair and was used to transform the TNO2A25 strain. Transformants were selected on MM containing urea as nitrogen source in the absence of uracil and uridine.

The thiamine repressible promoter was originally described in *A. oryzæ,* (Shoji *et al*., 2005) and has been used successfully to create conditional near-null mutants in a number of genes (*ssnF*, *azgA*, M. Mathieu, A. Rincón A., and C. Scazzocchio unpublished; *saltA*, Calcagno-Pizarelli *et al*., 2011). The cassettes containing the *thiA*^{p}*::ckiA, thiaA*^{p}*::FLAG-ckiA* and the *AFriboB-thiA*^{p}*::gfp::ckiA* fusions (in these constructions the 670 bp upstream of the *ckiA* gene were substituted by the *thiA* promoter) were constructed by sequential cloning of the corresponding PCR-amplified fragments in the *ApaI*, *SphI*, *EcoRI* and *NdeI* sites of the polylinker of the pGEM-T Easy vector (Promega) using primer pairs carrying restriction enzyme adaptors (Table S4). The *ckiA* 1256 bp upstream region (starting at 285961 of contig 1.78), the *ckiA* 831 bp partial ORF region (starting at 287886 of contig 1.78) and the 981 bp *thiA* promoter region (*thiA*^{p}) (*thiA*^{p}; 78472-79455 of contig 1.64), were, first, amplified using as template genomic DNA of TNO2A7 and the primer pairs CkiA 5' *ApaI* F-CkiA 5' *SphI* R, CkiA *EcoRI* F-CkiA *NdeI* R and ThiA^{p} *SphI* F-ThiA^{p} *EcoRI* R respectively. Subsequently, they were digested and cloned into the pGEM-T Easy vector, resulting in plasmid pT^{P}CkiA. For the *thiaA*^{p}*-FLAG-ckiA* cassette construction, the *thiA*^{p} fragment of plasmid pT^{P}CkiA was replaced by one carrying the FLAG tag sequence in addition, amplified using primer pairs ThiA^{p} *SphI* F-ThiA^{p} FLAG *EcoRI* R (pT^{P}FLAG-CkiA). For the *AFriboB-thiA*^{p}*::gfp::ckiA* cassette construction, the *AFriboB* amplified from plasmid p*nirA*Δ using the primer pair AFriboB *Sph*I F-AFriboB *Sph*I R and the *gfp* amplified from plasmid pERE-*nirA*^{c}*1*-GFP using the primer pair GFP *EcoRI* F-GFP *EcoRI* R were cloned at the *SphI* and *EcoRI* sites of plasmid pT^{P}CkiA respectively (pT^{P}GFP-CkiA). In the above plasmids the additional sequences of GCATGC (*SphI*) and GAATCC (*EcoRI*) are inserted at both sides of the *thiA* promoter sequence, resulting in a Glu-Ser linker prior the *ckiA* translation initiation codon of the *thiaA*^{p}*-FLAG-ckiA* and the *AFriboB-thiA*^{p}*::gfp::ckiA* cassettes. The resulting

transformation cassettes were amplified by the primer pair CkiA 5' *ApaI* F-CkiA *NdeI* R and used to either directly restoring full *ckiA* function by selecting on MM with glutamate as sole nitrogen source in the AMC314 strain, while introducing the thiamine promoter sequence and eliminating 670 bp of the *ckiA* putative promoter region (287216-287886 of contig 1.76) or complementing the *riboB2* auxotrophic mutation of strain TNO2A7 by selecting on MM media with urea as sole nitrogen source, in the absence of riboflavin. The intact single copy *in-locus* replacements were confirmed by Southern blot analysis by digesting genomic DNA with *NarI* using the PCR product of the primer pair CkiA *EcoRI* F-CkiA *NdeI* R as a probe.

Northern blot analyses: To extract RNA from ungerminated conidia, spores were harvested and suspended in 50 ml of 0.01 % Tween-80 solution. The suspension was filtered twice through blutex tissue to remove traces of mycelia and spores were collected on a Millipore filter (d=2.5 μm) using a vacuum pump. To monitor *ckiA* mRNA steady state levels during germination, CS2498 was grown for 3, 6 and 12 h in supplemented MM at 37° C in the presence of ammonium or glutamate as nitrogen source. To monitor *agtA* mRNA steady state levels in germinated conidiospores, strains were grown in supplemented MM at 37°C in the presence of urea as nitrogen source. Spores were harvested and suspended in 20 ml of 0.01 % Tween-80 solution. The suspension was filtered through blutex tissue to remove traces of mycelia. Germinated conidia developed after 4 h and 4 h 40min of incubation for CS2498 (wild type strain) and CS1901 respectively. They were collected by filtration on a Millipore filter (d=2.5 μm). The actin gene probe corresponds to a 2.5 kb *KpnI-BamHI* fragment of plasmid pSF5 and the *18S rRNA* probe to a 1.1 kb *EcoRI-EcoRI* fragment purified from plasmid pRG3. *agtA* mRNA steady state levels were monitored by hybridizing with a probe corresponding to the purified ~1.65 kb *NotI-NotI* restriction fragment from plasmid pAB1. *ckiA* mRNA steady state levels were monitored by hybridizing with a probe corresponding to a purified 706 bp PCR product obtained using the primer pair CKNF-CKNR and genomic DNA from strain CS2498 as template.

Protein manipulations: To analyse total protein extracts, cultures of strain VIE051 were grown on

supplemented MM at 30° C for 14 h in the presence of ammonium or γ-aminobutyric acid (GABA) as nitrogen sources. Mycelia were harvested and transferred to supplemented MM containing thiamine to a final concentration of 10 μM and GABA or urea as nitrogen sources and then incubated for additional 2-4 h. Sample concentrations were estimated by Bradford. Protein samples (30 μg) were fractionated on 10 % SDS-polyacrylamide gels and electroblotted (Mini Protean Tetra cell® BioRad) on PVDF membranes (Macherey-Nagel) for immunodetection. Membranes were treated with 3 % BSA, and immunodetection was performed using a mouse anti-GFP monoclonal antibody (Roche), a mouse anti-actin monoclonal (C4) antibody (MP Biomedicals), a mouse ANTI-FLAG® M2 monoclonal antibody and a secondary goat anti-mouse IgG (HRP)-linked antibody (Cell Signaling). Blots were developed on films (Fuji Super RX) by the chemiluminescent method using West Pico SuperSignal reagent (Pierce).

Membrane protein extracts were prepared from strains LH121, LH127, CAM13 and AMC264 (Calcagno-Pizarelli *et al.*, 2007). Conidiospores were inoculated in an appropriately supplemented MM containing ammonium L (+) tartrate as nitrogen source and incubated for 14 h at 30° C. Mycelia were harvested and transferred to supplemented MM containing 10 mM GABA (inducing conditions) or 10 mM ammonium (repressing conditions) and incubated for an additional 3 h at 37°C. Total membrane protein was determined using a bicinchoninic acid protein assay kit (Sigma). Samples were loaded onto 12% SDS-polyacrylamide gels (Bio-Rad) adjacent to Rainbow molecular mass markers (10,000 to 250,000 Da; Amersham Biosciences). Proteins were transferred onto nitrocellulose membranes that were reacted with rat anti-HA primary antibodies (catalog no. 3F10; Roche). The secondary antibody was a peroxidase-coupled goat anti-rat immunoglobulin G (Southern Biotechnology). Peroxidase activity was revealed using an ECL Western blotting detection system (Amersham Pharmacia).

Amino acid uptake assays: Four strains (CS2498, CAM45, CS1947 and CS1903) were assayed for proline uptake and of two strains (CS2498 and CS1947) for glutamate uptake. ^{3}H-labeled amino acid uptake was measured in germinating conidia as described in Robinson *et al*. (1973) and Tazebay *et al*. (1995), modified as follows. Conidiospores were inoculated in supplemented liquid MM using urea as

nitrogen source and incubated under agitation at 37° C. At the onset of germ tube appearance (4 h for *ckiA+* strains and 4 h 40 min for *ckiA-* strains), conidia were collected by filtration on a Millipore filter (d=2.5 μm) using a vacuum pump and re-suspended in 1 ml supplemented MM with urea as nitrogen source and then transferred on ice to prevent further growth. After pre-incubation of 90 μl of a germinating conidia suspension for 10 min at 37° C, 10 μl "Hot Solution" was added and the samples were incubated at 37° C for different time intervals (1, 2, 3, 4, 5 and 6 min). The reaction was stopped by the addition of 1 ml "Cold Solution" and transferred on ice for 5 min. Conidia were pelleted and washed with 1 ml of Wash Solution. Radioactivity was measured in sediment using a liquid scintillation counter (1209 Rackbeta, Wallac). Initial uptake rates were expressed in pmol of substrate incorporated per 1 min per 10^8 of viable conidia. The reported results for each type of uptake assay represent the mean values of three independent experiments. Hot Solution: supplemented MM with urea as sole nitrogen source containing 0.5 μCi of either L-[2,3-^{3}H] Proline (Amersham Pharmacia Biotech) or L-[G-^{3}H]Glutamic Acid (Amersham Pharmacia Biotech). The final concentration of hot and cold amino acids was 100 μM (5 x Km of proline transport by PrnB, Tazebay *et al.*, 1995) for proline and 540 μM (3 x Km of glutamate transport by the acidic amino acids transport system, Robinson *et al.*, 1973) for glutamate. Cold solution: supplemented MM with urea as sole nitrogen source and 1 mM of L-proline or 3 mM of L-glutamate. Wash Solution: supplemented MM with urea as sole nitrogen source and 10 mM of either proline or glutamate. Scintillation liquid: 667 ml of Toluene Scintillator (Packard Bioscience) and 333 ml of Triton-X100 (SIGMA).

Fluorescence microscopy: For fluorescent imaging of AgtA-GFP in the *ckiA*$^-$ mutants, uncoated glass bottom culture dishes were used (*MatTek* Corporation). For image acquisition and processing, a Zeiss Axiovert 200 inverted microscope with a fully motorised stage, offering a full incubation chamber with temperature control. An Improvision Volocity acquisition software was used. Stacks images were acquired using 63x objective 1.4 numerical aperture with a highly sensitive 1300x1000 pixel camera *Hamamatsu C4742-95-12ERG*. Zeiss; set 38 (BP 450-490nm bandpass excitation) for GFP and filter set 01 (BP 359-

371 nm bandpass excitation) were used to detect CMAC. Maxima projections of z-series stacks were made with Volocity and ImageJ software. Deconvolution of images was carried out using the Huygens software from SVI (Scientific Volumen Imaging, The Netherlands). For other microscopic observations, an Axioplan Zeiss phase-contrast epifluorescence microscope with appropriate filters was used and the resulting images acquired with a Zeiss MRC5 driven by AxioVs40 (version 4.40.0) software were then processed with Adobe Photoshop CS2 (version 9.0.2) software. Observation of asexual compartments was according to Pantazopoulou *et al.* (2007). Sample preparation and vacuolar staining with CMAC (7-amino-4-chloromethyl coumarin) (Molecular Probes) was according to Gournas *et al*. (2010).

Bioinformatic tools and databases: The following databases were consulted: For *A. nidulans*: Aspergillus Comparative Database (Broad Institute, http://www.broad.mit.edu/annotation/genome/aspergillus_terreus/MultiHome.html. *A. nidulans* Linkage Map (compiled by J.A. Clutterbuck, http://www.gla.ac.uk/acad/ibls/molgen/aspergillus/index. html). *Aspergillus* genome database, http://www.aspgd.org/. Other fungi: http://www.broadinstitute.org/scientific-community/data, http://www.jgi.doe.gov/genome-projects/, http://www.genolevures.org/yeastgenomes.html, http://www.yeastgenome.org/, *S. cerevisiœ* GFP fusions: http://yeastgfp.yeastgenome.org/ http://old.genedb.org/genedb/pombe/, http://www.candidagenome.org/, http://mycor.nancy.inra.fr/IMGC/TuberGenome/, http://fungalgenomes.org/wiki/Fungal_Genome_Links, http://podospora.igmors.u-psud.fr/, other organisms: http://www.ncbi.nlm.nih.gov/guide/proteins/. Blasts: above databases and http://blast.ncbi.nlm.nih.gov/Blast.cgi. Transporter proteins: http://www.membranetransport.org/ Protein alignments: http://www.ebi.ac.uk/Tools/msa/muscle/ and http://tcoffee.vital-it.ch/cgi-bin/Tcoffee/tcoffee_cgi/index.cgi, visualised with box shade: http://www.ch.embnet.org/software/BOX_form.html, Protein modelling was carried out with Swiss Pdb viewer (http://www.expasy.org/spdbv/, Guex and Peitsch, 1997) and http://zhanglab.ccmb.med.umich.edu/I-TASSER/, Zhang *et al*., 2008; Roy *et al*., 2010, and visualised with VMD (http://www.ks.uiuc.edu/Research/vmd/. Phylogenetic trees were obtained using:

http://www.phylogeny.fr/version2_cgi/alacarte.cgi (Dereeper *et al*., 2008). Aligned proteins were analysed for conserved surface or buried residues at: http://consurf.tau.ac.il/index_proteins.php.

Accession numbers. We show below the accession numbers of all genes included in Fig. 4. Mammalian homologues, NCBI reference sequences: α *M.m.* NM_146087.2; γ1 *M.m.* NM_173185.2; γ2 M.m. NM_001159591.1; γ3 M.m. NM_152809.2; δ (isoform 1) *M.m.* NM_139059.2, ε M. m. NM_013767.6; β *B.t.* NM_001098159.1; δ (isoform 1) *R.n.* NM_139060.3, Fungal homologues: *A. nidulans* (this article), *N. crassa*, *S. pombe* and *S. cerevisiæ* characterised homologues are referred to by their standard genes names. Other fungal homologues, referred by specific accession numbers: *C.a.* A orf193476CA, *C.a*.B1 orf197001CA; *C.a*.B2 orf192222CA; *Y.l*.A YALI0F08305; *Y.l*.B YALI0E26609, *C.i*.A1 CIMG01817; *C.i*.A2 CIMG03159; *C.i*.B CIMG04038; *F.o*.A FOXG03065; *F.o*.B FOXG05428; *S.s*.A SS1G09429; *S.s*.B SS1G03818; *S.n*.A SNOG00322; *S.n*.B SNOG05017; *T.m*.A GSTUMT00002552001; *T.m*.B GSTUMT00011786001.

Supplementary references

Bernreiter, A., Ramon, A., Fernandez-Martinez, J., Berger, H., Araujo-Bazan, L., Espeso, E. A., *et al.* (2007) Nuclear export of the transcription factor NirA is a regulatory checkpoint for nitrate induction in *Aspergillus nidulans*. *Mol Cell Biol* **27:** 791-802.

Delcasso-Tremousaygue, D., Grellet, F., Panabieres, F., Ananiev E. D. and Delseny M. (1988) Structural and transcriptional characterization of the external spacer of a ribosomal RNA nuclear gene from a higher plant. *Eur J Biochem / FEBS* **172:** 767-776.

Dereeper, A., Guignon, V., Blanc, G., Audic, S., Buffet, S., Chevenet, F., *et al.* (2008) Phylogeny.fr: robust phylogenetic analysis for the non-specialist. *Nucleic Acids Res* **36:** 465-469.

Fidel, S., Doonan, J. H. and Morris, N. R. (1988) *Aspergillus nidulans* contains a single actin gene which has unique intron locations and encodes a gamma-actin. *Gene* **70:** 283-293.

Guex, N. and Peitsch, M. C. (1997) SWISS-MODEL and the Swiss-PdbViewer: an environment for comparative protein modeling. *Electrophoresis* **18:** 2714-2723.

Pantazopoulou, A., Lemuh, N. D., Hatzinikolaou, D. G., Drevet, C., Cecchetto, G., Scazzocchio C., and Diallinas, G. (2007) Differential physiological and developmental expression of the UapA and AzgA purine transporters in *Aspergillus nidulans*. *Fungal Genet Biol* **44:** 627-640.

Roy, A., Kucukural A., and Zhang Y. (2010) I-TASSER: a unified platform for automated protein structure and function prediction. *Nature Protoc* **5:** 725-738.

Schinko, T., H. Berger, W. Lee, A. Gallmetzer, K. Pirker, R. Pachlinger, *et al.* (2010) Transcriptome analysis of nitrate assimilation in *Aspergillus nidulans* reveals connections to nitric oxide metabolism. *Mol Microbiol* **78:** 720-738.

Shoji, J. Y., Maruyama, J., Arioka M. and Kitamoto, K. (2005) Development of *Aspergillus oryzae* thiA promoter as a tool for molecular biological studies. *FEMS Microbiol Lett* **244:** 41-46.

Szewczyk, E., Nayak, T., Oakley, C. E., Edgerton, H., Xiong, Y., Taheri-Talesh, N. (2006) Fusion PCR and gene targeting in *Aspergillus nidulans*. *Nature Protoc* **1:** 3111-3120.

Yang, L., L. Ukil, A. Osmani, F. Nahm, J. Davies, C. P. De Souza, X. Dou, A. Perez-Balaguer, and S. A. Osmani (2004). Rapid production of gene replacement constructs and generation of a green fluorescent protein-tagged centromeric marker in *Aspergillus nidulans*. *Eukaryot Cell* **3:**1359–1362.

Yu, J. H., Hamari, Z., Han, K. H., Seo, J. A., Reyes-Dominguez, Y. and Scazzocchio, C. (2004) Double-joint PCR: a PCR-based molecular tool for gene manipulations in filamentous fungi. *Fungal Genet Biol* **41:** 973-981.

Zhang, Y. (2008) I-TASSER server for protein 3D structure prediction. *BMC Bioinformatics* **9:** 40.
